# Unveiling the research advances of sepsis: pathogenesis, precise intervention and clinical perspective

**DOI:** 10.1097/JS9.0000000000002668

**Published:** 2025-06-20

**Authors:** Lingxia Cheng, Yu Cao, Shihao Liu, Lukai Lv, Jianjun Zhang, Ji Bao, Guan Wang, Ping Xu

**Affiliations:** a Emergency Department, Zigong Fourth People’s Hospital, Sichuan, China; b State Key Laboratory of Biotherapy and Cancer Center, Collaborative Innovation Center of Biotherapy, West China Hospital, Sichuan University, Sichuan, China; c Innovation Center of Nursing Research, Nursing Key Laboratory of Sichuan Province, Key Laboratory of Transplant Engineering and Immunology, NHC, West China Hospital, Sichuan University/West China School of Nursing, Sichuan University, Sichuan, China; d Institute of Medical Big Data, Zigong Academy of Artificial Intelligence and Big Data for Medical Science,Sichuan, China

**Keywords:** Gram-negative bacteria, pathogenesis, precision treatment, sepsis, sepsis subtype

## Abstract

Sepsis is a life-threatening multi-organ dysfunction caused by the dysregulated systemic inflammatory and immune responses in the host to an infection. Despite continuous advances in the treatment of sepsis, its high morbidity and mortality seriously challenge global public health. Symptomatic treatments are currently applied to sepsis patients, while precise treatments acting on the individualized etiological and pathogenic factors are scant. To address the issue, the present review aims to illustrate the pathogenic mechanisms of Gram-negative bacteria, the immune imbalance of co-existing continuous inflammation and immunosuppression, and the increased susceptibility resulting from the imbalanced gut microbiota. Moreover, we summarized the therapeutic strategies for sepsis and the development of precise treatment acting on sepsis patients’ individualized subphenotypes and immune statuses. From the perspectives of etiological factors, pathogenesis, and precision treatment, we provide new insights into the future treatment of sepsis.

## Introduction

In 1991, the definition of sepsis was established (sepsis 1.0), defined as the systemic inflammatory response syndrome (SIRS) triggered by infection. Within this framework, more severe cases are termed severe sepsis (sepsis with organ dysfunction) and septic shock (sepsis with refractory hypotension). Sepsis 3.0 was proposed in 2016 as a life-threatening organ dysfunction caused by a dysregulated host response to infections^[1,2]^, and severe sepsis is no longer cited^[3]^. Globally, there were 48.9 million patients who suffered from sepsis in 2017^[4]^. The mortality of sepsis in any country may be seriously underreported, and its incidence is growing elevated as the population aging^[1,5]^. Although the mortality of sepsis decreased by 52.8% from 1990 to 2017, the 11 million sepsis-related deaths annually accounted for an astonishing 19.7% of global deaths^[4]^. Moreover, recurrences, secondary damage, and sequelae of sepsis after discharge seriously harm human health^[6-9]^. It is reported that 31.5% of survivors of sepsis rely on nursing care after discharge, resulting in a huge medical expense of 29 000 euros for each survivor within the 3 years of onset. An estimated healthcare burden of global survivors of septic shock is inflated to $24 billion per year. Sepsis imposes a serious burden on society and families worldwide^[10,11]^.

**HIGHLIGHTS**

- Sepsis is a life threatening syndrome initiated by microorganisms, characterized by significant heterogeneity.
- Understanding the mechanisms of bacterial infection, the host’s excessive inflammatory response and immune suppression, as well as the mechanisms by which dysregulation of the gut microbiota leads to susceptibility, is necessary.
- “One-size-fits-all” strategies to treat sepsis are no longer satisfactory.
- Host directed therapy (sepsis subtypes and immunotherapy) has potential roles in the treatment of sepsis.
- It is hoped that from the perspective of pathogenic factors, pathogenesis, and personalized treatment, new insights can be provided for the clinical precision treatment and multimodal combination treatment of sepsis.

Infection is the only cause of sepsis. The source of sepsis may come from any infected part of the body. Multiple pathogens can be detected in one hospitalized sepsis patient. In 2019, more than 6 million patients died from three infectious syndromes, including 2 million from lower respiratory tract and blood infections, and 6 million from peritoneal and intra-abdominal infections^[12]^. Particularly, 400 000 deaths are attributable to and 1.5 million deaths are associated with lower respiratory tract infections^[13]^. The global impact of antimicrobial resistance (AMR) is profound. Computational predictive modeling for 2019 estimated that 1.27 million deaths were directly attributable to bacterial AMR globally, establishing AMR as the third leading underlying cause of death that year, following ischemic heart disease and stroke^[13]^. Furthermore, bacterial AMR was associated with an estimated 4.95 million deaths in the same period, underscoring its vast global health impact. Lower respiratory tract infections were a major contributor to this burden, accounting for over 1.5 million deaths associated with AMR in 2019. Pathogens such as *Escherichia coli, Klebsiella pneumoniae, Acinetobacter baumannii*, and *Pseudomonas aeruginosa* have emerged as key contributors to these resistance-associated deaths, with *E. coli* being particularly predominant^[13]^. Given that sepsis is a common and life-threatening manifestation of severe infections, frequently complicated by AMR – the rise of antimicrobial resistance has critically escalated the challenge of managing septic patients and preventing sepsis-related mortality worldwide^[14]^. Thus, antimicrobial resistance represents a rapidly aggravating global issue, with resistance-complicated sepsis at the forefront of this crisis.

Pathogens enter the human body through the skin, mucous membranes, or other routes, colonize at the site of infection, and may produce toxins or enzymes that destroy tissues and facilitate the spread of the pathogen. Throughout this process, after bacteria and toxins enter the body, they activate the host’s immune cells, producing and releasing a large amount of inflammatory mediators (such as TNF-α, IL-1β, IL-6, etc.), causing local or systemic inflammatory responses. If the immune system fails to control the infection, the body develops excessive inflammatory responses and shifts into a state of immunosuppression, leading to sepsis. As sepsis progresses, the initial hyperinflammatory response may transition into a state of immunosuppression. Excessive inflammatory responses and immunosuppression are not mutually exclusive; they can transform into each other during sepsis, forming a dynamic change in immune status. In the progression of sepsis, gut commensal microbes also play a significant role. Dysbiosis can lead to increased disease susceptibility. The disruption of the gut microbiota and the impairment of the intestinal barrier in sepsis patients can result in the translocation of opportunistic pathogens and their metabolic products, which can further affect the function of immune cells in the body, leading to or exacerbating immunological dysregulation.

In a study that included 8135 patients with suspected or confirmed infections, 67% had Gram-negative bacteria detected, making Gram-negative bacteria the main causative agent^[15]^. This text focuses primarily on Gram-negative sepsis. A proper anti-inflammation against pathogen infections stimulates tissue repair and balances homeostasis^[16]^. An overwhelming inflammation, nevertheless, induces immune dysregulation and eventually organ dysfunction^[16]^. The intensity and extent of the inflammatory response vary according to the type, load, and virulence of the pathogen, as well as the underlying disease, immune status, age, and nutritional status of hosts^[17]^. Individualized conditions of pathogens and physical status result in the heterogeneity of sepsis. In response to infections, an inflammatory cascade alters coagulation, complement, and microbiome systems, causes extensive damage to endothelial cells, coagulation and fibrinolysis, and ultimately induces septic shock and disseminated intravascular coagulation (DIC). So far, a clear illustration of the heterogeneity of sepsis has not been fundamentally unveiled.

A one-size-fits-all approach is no longer satisfying the treatment of sepsis due to the heterogeneity. The current standard of care for treating sepsis involves managing the infection sites through drainage, administering antibiotics, providing fluid resuscitation, and providing supportive care for failing organs^[18]^. According to the statistics from more than 200 relevant randomized controlled trials (RCTs) within the past 3 decades, sepsis patients are poorly responsive to the treatment regimens.

A concept of sepsis bundle was proposed by the Surviving Sepsis Campaign (SSC) to act on the treatment of sepsis, but as understanding of sepsis continues to deepen, higher demands are placed on treatment^[18]^. Furthermore, to address the issue of sepsis heterogeneity, numerous scholars have utilized genomics, transcriptomics, proteomics, and other omics approaches along with clinical data to classify sepsis^[19]^, dividing it into different subgroups based on treatment response, outcomes, shared clinical and laboratory features of two or more, or specific pathophysiological changes^[20,21]^. A clear illustration of the pathogenesis of different subtypes of sepsis contributes to achieving the precise treatment. In the present article, we summarize infection mechanisms, immune dysregulation (persistent inflammatory response and immune suppression), and gut microbiota (increased susceptibility). Besides, conventional and newly emerging therapeutic strategies for sepsis were summarized, especially the individualized, precise treatment based on the subtype and immune status.

## Materials

### Literature search strategy

This review aimed to comprehensively gather current research on the pathogenesis, precise interventions, and clinical perspectives of sepsis. A systematic search was conducted across multiple electronic databases, including PubMed, Scopus, Web of Science, and Google Scholar. The search period was defined from January 2000 to December 2024 to ensure the inclusion of both foundational studies and the latest advancements in the field.

The search strategy combined free-text terms and subject headings (e.g., MeSH terms in PubMed). Core search terms included, but were not limited to: “sepsis”, “septic shock”, “pathogenesis”, “etiology”, “Gram-negative bacteria”, “Escherichia coli”, “Klebsiella pneumoniae”, “Pseudomonas aeruginosa”, “Acinetobacter baumannii”, “immune response”, “immunosuppression”, “inflammation”, “cytokines”, “neutrophils”, “endothelial dysfunction”, “coagulation”, “complement system”, “gut microbiota”, “dysbiosis”, “precision medicine”, “sepsis subtypes”, “sepsis biomarkers”, “sepsis treatment”, and “immunotherapy”. These keywords were combined using Boolean operators (AND, OR) to optimize the specificity and sensitivity of the retrieved results.

### Literature screening was based on the following criteria

Inclusion Criteria: Original research articles (both experimental and clinical studies), high-quality review articles, meta-analyses, and guidelines from recognized academic institutions and professional societies were primarily included. The focus was on literature relevant to the etiology, pathogenesis of sepsis (particularly concerning Gram-negative bacteria), immune dysregulation (such as the coexistence of persistent inflammation and immunosuppression), gut microbiota dysbiosis leading to increased susceptibility, clinical diagnosis, and therapeutic strategies (including individualized precision treatment based on subtypes and immune status).

Exclusion Criteria: Non-English language publications, duplicate articles, conference abstracts, letters to the editor, and articles not directly relevant to the scope of this review were excluded. Case reports were generally not included unless they were highly illustrative of a specific point.

Two investigators independently performed literature screening and data extraction. Initial screening was conducted by reviewing titles and abstracts, followed by a full-text assessment of potentially eligible articles to determine final inclusion. Any discrepancies were resolved through discussion or consultation with a third expert if necessary. Relevant data on pathogenic mechanisms, immune responses, organ dysfunction, diagnostic markers, existing and emerging therapeutic strategies (especially precision treatments and immunotherapies) were extracted and synthesized narratively.

Although this is primarily a narrative review, the principles of the PRISMA (Preferred Reporting Items for Systematic Reviews and Meta-Analyses) guidelines were referred to during the literature search and selection process to enhance comprehensiveness and transparency. During the conception and writing of the review, no artificial intelligence assistance was used to avoid the loss of authenticity in the content^[22,23]^.

## Gram-negative

Sepsis can be caused by various pathogenic microorganisms like bacteria, fungi and parasites, of which bacterial infections are the predominant cause (>90%). Bacterial infections are categorized into Gram-positive and Gram-negative, and the latter is more common (67%, *n* = 3540)^[15]^. The incidence of severe sepsis and relative levels of inflammatory factors (e.g., CRP, PCT, TNF-α) are both higher in patients with Gram-negative sepsis than those of Gram-positive sepsis^[19]^, alongside the higher prevalence^[20]^, longer length of stay^[21]^, higher mortality^[20]^ and higher risk of drug resistance^[24]^. Gram-negative sepsis has become a priority to be concerned about in clinical practice.

In an observational study involving 1150 centers, it was found that 67% of cultured microorganisms were Gram-negative bacteria, 37% were Gram-positive bacteria, and 16% were fungi^[15]^. *Staphylococcus aureus, E. coli, Streptococcus pneumoniae, K. pneumoniae*, and *P. aeruginosa* isolated from the samples accounted for 54.9% of the fatal bacterial species^[25]^. Clinical practice must pay attention to *A. baumannii*, which has a multidrug resistance rate as high as 71%^[25,26]^. Drug resistance is a significant barrier to the clinical management of sepsis, increasing mortality. Gram-negative bacteria have become the most common pathogens causing sepsis. Among them, *E. coli, K. pneumoniae*, and *P. aeruginosa* are the main pathogens causing Gram-negative infections, ranking in p 10. *A. baumannii* has become a long-term challenge for clinicians due to its drug resistance. Therefore, this review summarizes the infection and antimicrobial resistance mechanisms of *E. coli, K. pneumoniae, P. aeruginosa*, and *A. baumannii.*

### Escherichia coli: epidemiology and pathogenicity

*Escherichia coli* strains are the most common causes of infection, and their case fatality rate ranges from 5% to 30%^[27-29]^. *E. coli* pathogens mainly cause urinary tract infections and gastrointestinal infections (Table 1)^[28,29]^. Even though urinary tract infections serve as the leading cause, their mortality is lower than that caused by other sources of *E. coli* sepsis^[28]^.

**Table 1:** Comparison of common Gram-negative bacteria in sepsis

Category	*Escherichia coli*	*Klebsiella pneumoniae*	*Pseudomonas aeruginosa*	*Acinetobacter baumannii*
Common infection sites	Urinary tract, abdominal cavity, bloodstream	Pneumonia, urinary tract, sepsis	Hospital-acquired pneumonia, burn infections	ICU pneumonia, trauma infections, bloodstream
Major virulence factors	Adhesins, endotoxins	capsules, LPS, siderophores, pili and allantoin	Outer membrane proteins, toxins, biofilm	Biofilm formation, capsule
Resistance mechanisms	ESBLs, aminoglycoside-modifying enzymes	KPC, NDM, OXA-type enzymes, ESBLs, carbapenem	Outer membrane proteins, transcriptional regulators, efflux pumps, β-lactamases, biofilms	Enzymes, increased efflux
Common resistance genes	blaCTX-M, blaTEM, blaSHV	blaKPC, blaNDM,	blaVIM, blaIMP, mexAB-oprM	adeABC efflux pump
Biofilm formation ability	Moderate	Strong	Strong	Very strong
Clinical impact/risk	Common in community infections, ESBL producers linked to high mortality	High resistance rates, carbapenem-resistant strains increase nosocomial mortality	Difficult to treat, leads to chronic and recurrent infections	Extremely drug-resistant, limited treatment options, high ICU mortality
Resistance trend	Increasing ESBLs, declining carbapenem susceptibility	Rapid spread of carbapenemases like KPC and NDM	Inherently resistant, rising carbapenem resistance	Persistently high MDR, growing XDR prevalence

ESBL, extended-spectrum β-lactamase; ICU, intensive care unit; IMP, imipenem; LPS, lipopolysaccharide; KPC, *Klebsiella pneumoniae* carbapenemase; MDR, multidrug-resistant; XDR, extensively drug-resistant; NDM, New Delhi metallo-β-lactamase; OXA, oxacillinase; VIM, Verona integron-encoded metallo-β-lactamase.

*E. coli* strains are grouped into four phylogenetic groups: A, B1, B2, and D. Group A represents commensalism and group B represents pathogenic clones^[30]^. More virulence factors, such as toxins, adhesins, and siderophores, that support initial site invasion, dissemination, and bloodstream survival are expressed by groups B2 and D^[31,32]^. Capsular polysaccharide O-acetylation is engaged in the initial infection and immune evasion in the bloodstream by escaping Siglec-mediated innate immunity and lysosomal degradation^[33,34]^. The serine protease Pic secreted by *E. coli* is an important virulence factor that allows pathogens to colonize and survive in the bloodstream and multiple organs by directly cleaving the complement molecule C3/C3b. Serine protease is essential for the adaptability of pic-encoded *E. coli* strains. It triggers white cell migration via targeting leukocyte adhesion-associated factors (CD43, CD44, CD45, and CD93), suppresses the complement system activation, and induces the production of massive inflammatory mediators, eventually leading to infections or even deaths^[35,36]^. Adhesins have a well-established role in the pathogenesis of *E. coli*. In mice with tail vein injection of *E. coli* strains, mutants lacking type I and P pili are outcompeted by the wild-type strains, indicating that pili adhesion contributes to local infection and systemic colonization^[37]^. Р pili expressed by uropathogenic *E. coli* bind to P antigens on red blood cells and play a potential role in the systemic transmission of bacteria^[38]^. A-hemolysin is an important pathogenic factor leading to urinary tract infection in a mouse model. It induces the release of pro-inflammatory factors (IL-1β, TNF-α and IL-6) by directly activating the immune system or indirectly activating it via stimulating the purinergic signaling pathway. Additionally, α-hemolysin induces platelet activation through the engagement of P2Y12 and P2Y1 receptors, contributing to the development of severe sepsis^[39]^.

Poly-*N*-acetylglucosamine is an extracellular *E. coli* polysaccharide that contributes to biofilm formation and extracellular matrix binding. Extracellular *E. coli* polysaccharide poly-*N*-acetylglucosamine aids in the production of biofilms and the binding of extracellular matrix^[40]^. By actively limiting iron bioavailability, the immune system of the host blocks pathogen growth. Siderophores are iron-chelating compounds to help the organism accumulate iron. In the isolated strains, the expression level of siderophores is significantly correlated with the pathogenicity of the B2 group isolates^[40]^. Siderophores allow bacteria to acquire iron in the blood, and overexpressed siderophores in *E. coli* isolates are conducive to iron absorption and bacterial survival^[41]^, making the host more susceptible to infection. The correlation between the genes required for P pili and the survival of *E. coli* remains controversial. PapC, papGII and/or papGIII that assemble P pili are protective factors^[28]^. On the contrary, *P pili* are essential for infection^[37]^ and UTI^[42]^ in mouse models, which are responsible for triggering the immune response to eliminate pathogens by the TLRs signaling pathway^[43,44]^.

Currently, antimicrobial resistance is a major concern in the treatment of *E. coli* infection. In southern Sweden, the proportion of *E. coli* infections resistant to fluoroquinolones rises dramatically from 8.4% to 13.6%, and the proportion resistant to third-generation cephalosporins rises from 4.9% to 7.3%^[45]^. A Finland study reported that the annual proportion of *E. coli* isolates producing extended-spectrum beta-lactamase (ESBL) in blood culture increases from 2.4% to 8.6%^[46]^. *E. coli* isolates usually carry antimicrobial plasmids encoding β-lactamase (bla), ESBL and carbapenemase^[47,48]^. *E. coli* sequence types (STs) 131, 193 and 410 are collectively responsible for a large proportion of *E. coli* infection^[31,49]^. Their isolates often carry plasmid-mediated resistance genes *CTX-M-14, CTX-M-15, CTX-M-27, CTX-M-55*, and *CTX-M-65* that transfer between bacteria through plasmids (Table 1)^[32,50,51]^. The annoying antimicrobial resistance greatly weakens the effectiveness of antibiotics against *E. coli* infections or even makes them ineffective.

### Klebsiella pneumoniae: epidemiology and pathogenicity

*Klebsiella pneumoniae* is an opportunistic pathogen that colonizes the upper respiratory tract and gastrointestinal tract. Nosocomial infections are the dominant cause of *K. pneumoniae*, accounting for 57%-70%^[52,53]^. *K. pneumoniae* usually affects middle-aged and elderly people with underlying diseases like diabetes mellitus, and its mortality is influenced by the strains, advanced age, immune deficiency, and potential comorbidities. The incidence of sepsis in survived and non-survived patients with *K. pneumoniae* ranges from 13%-26% and 54%-79% respectively^[54]^. The mortality of antibiotic-susceptible *K. pneumoniae* is lowered at 26%, which increases with the increasing antibiotic resistance.

Based on their virulence and resistance to carbapenem, *K. pneumoniae* are divided into two groups: classical *K. pneumoniae* (cKp) and *hypervirulent K. pneumoniae* (hvKp). cKp is the most common subtype of *K. pneumoniae* in Western countries that carries antimicrobial plasmids. hvKp infection was first reported in Southeast Asia. It is associated with community-acquired infections, liver abscesses, endophthalmitis, and meningitis^[55]^. The invasiveness of *K. pneumoniae* is related to high mucus viscosity^[56]^. Similar to *E. coli*, pathogenic factors of *K. pneumoniae* include capsules, lipopolysaccharide (LPS), siderophores, pili and allantoin (Table 1). Differently, *K. pneumoniae* lacks specific toxins like hemolysin or cytotoxic necrotizing factors. Capsules are intrinsic virulence factors of *K. pneumoniae* that allow immune evasion by protecting the bacteria from phagocytosis and serum resistance^[57,58]^. The capsule primarily promotes the survival of *K. pneumoniae* by evading capture by macrophages^[59]^. Endotoxin, or lipopolysaccharide, is the primary component of Gram-negative bacteria’s outer membrane that induces sepsis. LPS stimulates the release of pro-inflammatory factors (e.g., TNF-α, IL-6 and IL-1β) by activating immune cells (e.g., monocytes, macrophages, neutrophils and natural killer cells) via TLR4. Activated granulocytes by inflammatory cells further induce a series of events to aggravate inflammatory responses, including endothelial cell damage, platelet adhesion, and accumulation of oxygen free radicals and lipid metabolites. The complicated reciprocal causation finally causes an inflammatory cascade^[60]^. Allantoin is a nitrogen source in bacteria, and it also provides nitrogen and carbon to *K. pneumoniae*. Allantoin increases the virulence of *K. pneumoniae* in liver infections by structural operons^[61]^.

Neutrophils and alveolar macrophages are essential to control the initial onset of *K. pneumoniae* lung infection and sepsis^[62]^. Host thrombospondin-1 (TSP-1) is an ECM necessary for the invasion of *K. pneumoniae* into the lungs and spleen. Inactivating neutrophil elastase, TSP-1 facilitates the survival of *K. pneumoniae* in the lungs during the early stage of infection. TSP-1^−/−^ mice present a higher bacterial clearance rate and lower bacterial load in the spleen than those of wild-type mice^[63]^. In addition, *K. pneumoniae* escapes from pyroptosis by upregulating IL-10 and thus survives from the killing of innate immune cells^[64]^. To evade immune cell killing, *K. pneumoniae* impairs or inactivates IL-10 or TSP-1. Virulence factors of *K. pneumoniae*, like siderophores, increase angiogenesis and vascular epithelial permeability by upregulating pro-inflammatory cytokines (e.g., TNF-α, IL-6 and IL-1β), providing an immune escape approach from the initial infection site (the lung) to the bloodstream infection. Catheter-related sepsis offers lower mortality than secondary sepsis due to the lower incidence of *hvKp* directly infected in the bloodstream^[65]^. Secondary sepsis is usually caused by pathogens evading the immune system by penetrating the epithelial barrier. Carbapenem-resistant (CR) *K. pneumoniae* is extremely concern for its high resistance rate and virulence. ESBL-producing and CR *K. pneumoniae* are predominantly popular in Asia, with the incidence in China of 31%-37.8% (Table 1). Their onset is probably linked with the subspecies of *K. pneumoniae*^[66]^. ST11 CR hvKp was initially isolated in China in 2016, manifesting high virulence, multi-drug resistance and a high transmission rate. It directly induces severe infections, and more seriously, antibiotic resistance further poses a challenge in the treatment or even induces fatal events^[67]^.

### Pseudomonas aeruginosa: epidemiology and pathogenicity

*Pseudomonas aeruginosa* is the third most common Gram-negative bacterium isolated from clinical cases. In hospital environments, *P. aeruginosa* can colonize medical devices with the assistance of its binding factors like flagella, pili and biofilms^[68]^. As a result, *P. aeruginosa* infections typically occur in healthcare settings, leading to ventilator-associated pneumonia, intensive care unit (ICU) infections, central line-associated bloodstream infections, surgical site infections, urinary tract infections, burn infections, keratitis and otitis media (Table 1)^[69,70]^. Due to the high detection rate in patients with bronchiectasis and critically ill patients, the mortality of *P. aeruginosa* infection ranges from 32% to 62%^[71-73]^.

Classifications of *P. aeruginosa* are generally based on biology, serology and molecular biology, and the serotype classification is the established method. Serotype O11 is unique in producing glycosidases than other serotypes of *P. aeruginosa*, which is closely linked with nosocomial outbreaks and sepsis arising from drug abuse^[71,74,75]^. Serotype O11 is common in multidrug-resistant (78.4%) and ExoU-positive (89.2%) strains of *P. aeruginosa*^[71]^. ExoU is a phospholipase that destroys the integrity of the host membrane. It massively spreads from the lungs to the bloodstream^[76]^. The type III secretion system (T3SS) provides a virulence mechanism enabling pathogens to spread from the lungs to the bloodstream via directly injecting bacterial effector proteins into the host cell cytoplasm, thus increasing mortality in humans and animal models^[73]^.

*P. aeruginosa* produces three T3SS exotoxins: ExoU, ExoS, and ExoT. Among these, ExoU and ExoS are linked to high bacterial loads and *P. aeruginosa* infection mortality^[71,77]^. ExoS and ExoT directly help *P. aeruginosa* evade host immune cells by disrupting the actin cytoskeleton^[78,79]^. ExoS is specifically injected into neutrophils in early-stage *P. aeruginosa* pneumonia and into type I alveolar cells in the late stage^[80]^. Type I alveolar cells with ExoS form aggregates, and the abundance of dead cells increases as infection progresses^[78]^. Aggregate formation is associated with the transmission of *P. aeruginosa* from the lungs to the bloodstream. T3SS-negative P. aeruginosa secretes the toxin ExlA, which causes necrosis of the epithelium and myeloid cells. Lung invasion and hematogenous spread of *P. aeruginosa* in a murine pneumonia model are influenced by the effect of ExlA on disrupting the pulmonary vascular barrier^[81]^. In addition, the type II secretion system (T2SS) and LasB elastase are key virulent factors for *P. aeruginosa*. LasB increases vascular permeability through dissociating ECMs via interacting with vascular endothelial-cadherin^[82]^.

*P. aeruginosa* strains that produce only ExoU can destroy host cells directly by rupturing the cell membrane, whereas strains that express ExoS and ExoT use exotoxins to avoid being killed by early innate immune cells. ExoS even kills type I alveolar epithelial cells and destroys the epithelial barrier. ExoT cleaves ECMs and causes vascular leakage^[78,82]^. T3SS is required for the virulence of *P. aeruginosa* in a burning injury model^[83]^. *P. aeruginosa* is different from *K. pneumoniae* in that it expresses strong exotoxins that are comparable to hemolysin and cytotoxic necrosis factor made by *E. coli*.

*P. aeruginosa* is an opportunistic pathogen that is hardly eradicated if antimicrobial-resistant. Romania and Serbia (2015-2020) are the two countries suffering from the highest resistance rate of *P. aeruginosa*. Generally, drug resistance of *P. aeruginosa* can be intrinsic, acquired or adaptive^[84]^. Outer membrane proteins, transcriptional regulators, efflux pumps, enzymes, and biofilms play different roles in the antimicrobial resistance of *P. aeruginosa* (Table 1). The production of bla is the well-recognized machinery for *P. aeruginosa* resistance. For bla, it can be divided into cephalosporinase, high-level AmpC, ESBL and carbapenemase^[85,86]^. Genes encoding aminoglycoside-modifying enzymes (AMEs) in *P. aeruginosa* are responsible for the high rate of drug resistance^[87]^. AMEs can be divided into acetyltransferases (AACs), nucleotidyltransferases (ANTs) and phosphotransferases (APHs) that respectively initiate *N*-acetylation, adenylation and/or O-phosphorylation of aminoglycosides to end in a treatment failure^[88]^. Efflux transporters are the key cause of the multidrug resistance of *P. aeruginosa*^[89]^. Mobile genetic elements (MGEs) like integrons, plasmids, and transposons facilitate the rapid dissemination of clinically important genes^[90]^. The formation of bacterial biofilms protects bacteria against various chemical and environmental stresses (e.g., phagocytosis), leading to widespread drug resistance and potential infections^[91]^.

### Acinetobacter baumannii: epidemiology and pathogenicity

Numerous infections in the respiratory system, urinary system, wound areas, abdominal cavity, and neurological system can result from the skin, nasal cavity, and trachea being colonized by *A. baumannii* (Table 1). Respiratory infections are the most common cause (60%)^[92]^. *A. baumannii* is a major cause of nosocomial infections in recent years. It is reported that hospital-acquired infections account for 75% of *A. baumannii* infections, and they are responsible for 86% of drug-resistant strains^[93]^. The attributable mortality of community-acquired pneumonia and bloodstream infections caused by *A. baumannii* is up to 60% and 43%, respectively^[94,95]^. Severe complications developing in ICU patients with *A. baumannii* infections are primary drivers of the extremely high mortality rates. Hospital settings provide a favorable reservoir of *A. baumannii*^[96]^, and a longer length of stay predicts a higher risk of *A. baumannii*^[97]^. Usually, the same strains of *A. baumannii* are detected in nasal isolates and isolates from hospital environments^[98]^. The colonization rate in hospitalized patients remains higher, with the statistics of 54%-92% and 63% reported in Taiwan and the United States, respectively^[98]^. People may suffer from opportunistic infections after antibiotic treatment of *A. baumannii* infections^[96,99]^. Prevention strategies can significantly reduce the risk of *A. baumannii infection* and the disease burden^[92]^.

*A. baumannii* often results from secondary infections caused by contaminated healthcare devices and surgical site infections^[92,93,97]^. The high mortality of *A. baumannii* infection is linked with acquisitions and expressions of resistance genes, capsule production and biofilm formation (Table 1)^[100]^. Biofilms offer a favorable environment for the survival of *A. baumannii*, serving as the basis for host infections from contaminated healthcare devices. *A. baumannii* bloodstream infection downregulates genes for biofilm formation (e.g., fimA and ompA) and adhesion molecules, but upregulates motility-associated genes (e.g., PilQ, YebC, H-NS and ExbD)^[101,102]^. The lytic transglycosylase gene mltB is essential for the colonization of *A. baumannii* in the respiratory tract of pneumonia mice, although the exact mechanism leading to bacterial infections is not yet clearly elucidated^[103]^. *A. baumannii* strains are resistant to killing by normal human serum^[104]^. Regulatory mechanisms on immune escape and adhesion molecules are of significance to unveil the pathogenesis of *A. baumannii* infection. Loss of mltB impairs biofilm formation and adhesion to epithelial cells by disrupting pilus assembly. Meanwhile, mltB is involved in the serum resistance of *A. baumannii*^[103]^. Easily affected by physiological stresses, *A. baumannii* is more susceptible to oxidation, acidity and osmosis. The genes pntB, feoB and fepA allow the survival of *A. baumannii* by partially endowing it with antimicrobial peptide resistance^[104]^. The production of capsules is a protective mechanism, and genes required for capsule synthesis (e.g., wzc and gpi) are upregulated by serum stimulation^[101]^. Additionally, by binding plasminogen, a regulator of the complement cascade, *A. baumannii* inhibits the development of terminal complement complexes on the bacterial surface^[105]^. The above conditions contribute to the persistence of immune responses to *A. baumannii*. The intensity of immune responses varies in different strains^[104]^. Survival of *A. baumannii* in the bloodstream may be more dependent on its survival mechanisms and the ability to maintain bacterial burden in the blood rather than traditional virulence factors^[103,104,106]^. Transcriptomic analysis revealed that *A. baumannii* upregulates genes associated with capsule biosynthesis and siderophore during sepsis^[102]^. Through increasing the zinc availability in plasma via upregulating zinc transporters znuB and znuC and downregulating zur, the survival rate of *A. baumannii* is greatly elevated^[101]^.

Although *A. baumannii* is not the predominant pathogen leading to sepsis, great concerns have been raised about the multi-drug resistance issue^[107]^. In 2019, the Centers for Disease Control and Prevention (CDC) in the United States proposed that *A. baumannii* is a human threat due to the high resistance rate. In China, more than 45% of *A. baumannii* strains colonized in the lower respiratory tract are resistant to imipenem^[108]^ and globally, more than 71% of strains are multidrug-resistant (Table 1)^[109]^. In the United States, 27% of patients with mechanical ventilation are infected with multidrug-resistant *A. baumannii*^[110]^. A significant increase in the drug resistance rate of *A. baumannii* was found in Ethiopia from 2017 to 2022^[111]^.

- *baumannii* contains three RND-type efflux pumps, which play an important role in the survival within the host and the resistance to antimicrobial drugs^[112,113]^. It is known that the two-component system AdeRS regulates the AdeABC efflux pumps, which promote resistance to aminoglycosides, tetracyclines, fluoroquinolones, trimethoprim, chloramphenicol, β-lactams, and tigecycline (Table 1)^[112]^. AdeFGH efflux pumps, regulated by a LysR-type transcriptional regulator namely adeL, induce resistance to chloramphenicol, fluoroquinolones, trimethoprim, tetracycline, tigecycline, clindamycin and sulfonamides^[112]^. AdeIJK efflux pumps, regulated by a TetR-type regulator namely AdeN, favor the resistance to bla, chloramphenicol, tetracyclines, erythromycin, fluoroquinolones, fusidic acid, novobiocin, and trimethoprim^[112]^. In multicenter studies, 1% of *A. baumannii* isolates exhibited pan-drug resistance, and colistin was the only reliable antimicrobial agent left for treatment^[25,114]^.

## The pathogenic mechanisms of sepsis

**Figure 1.**
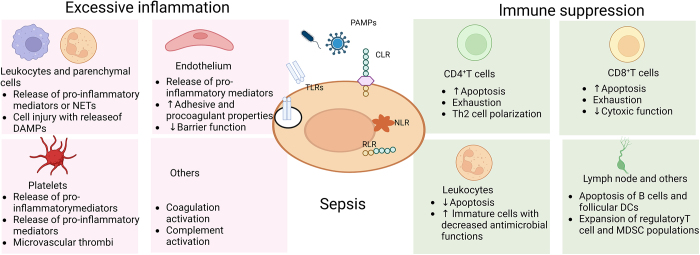
Host response to infection and sepsis. The host’s innate immune system is the first line of defense against the invading pathogen, building up a protective immunity (left). Leukocytes, immunological cells, and parenchymal cells, such as epithelial and endothelial cells, are involved in the early phases of the local immune response to the invader. They are activated by cell surface receptors and PRRs, including TLRs, NLRs (activation of NLRs forms inflammasomes), RLPs and CLRs. Protective immunity typically works well enough to eradicate the invasive pathogen by inducing pro-inflammatory reactions, which include phagocyte recruitment, complement and coagulation system activation locally, and the production of cytokines and chemokines. The outcome of a compensatory mechanism to stifle the initial inflammation and avoid tissue damage is rebalanced immunological homeostasis. However, when the invading pathogen prevails over the protective immunity, the imbalanced immune homeostasis eventually results in tissue damage and sepsis (right). PRRs pattern recognition receptors, TLRs Toll-like receptors, NLRs Nod-like receptors, RLPs RIG-I-like receptors, CLRs C-type lectin receptors.

The innate immune system is the first line of defense against invading pathogens. After pathogens enter the body, immune cells clear the pathogens through a series of complex mechanisms, including recognizing the pathogens, initiating inflammatory responses, phagocytosing and digesting the pathogens, and activating adaptive immune responses (Fig. 1). This process involves the coordinated action of various immune cells, including macrophages, neutrophils, T cells, and B cells. Immune cells’ pattern recognition receptors (PRRs) are in charge of identifying conserved motifs expressed by microbes as well as pathogen-associated molecular patterns (PAMPs). Receptors for PAMPs are positively expressed in subtypes of immune cells and parenchymal cells. Generally, the PRRs include Toll-like receptors (TLRs), nucleotide-binding oligomerization domain-like receptors (Nod-like receptors, NLRs), retinoic acid-inducible gene (RIG)-l-like receptors (RLRs), C-type lectin receptors (CLRs) and cytosolic RNA and DNA sensors^[115,116]^ (Fig. 1). After being recognized and interacting with PAMPs, PRRs initiate the upregulation of inflammatory genes, secretion of relevant factors, and the activation of innate immunity to eliminate pathogens and balance the immune homeostasis. Once pathogens evade the immune defense, continuous stimulation of PRRs impairs the machinery of the protective inflammatory response and eventually breaks the balance of immune homeostasis, leading to tissue damage and sepsis^[17,117]^. PRRs interact with damage-associated molecular patterns (DAMPs), which are linked to a vicious cycle of prolonged, excessive inflammation and cell damage, in addition to recognizing exogenous PAMPs (Fig. 1).

An inflammatory response in sepsis is mainly driven by inflammatory cells, cytokines, oxygen free radicals, complement and coagulation systems. A dynamic balance of the immune system is essential for immune response. Dysregulation of the immune system is a key pathogenic event in sepsis, leading to systemic inflammation, organ damage, immune suppression, or even death. Age is associated with innate immunity and cytokine signaling. Compared with those younger than 50 years of age, sepsis patients older than 70 years are prone to damage to the innate immunity and cytokine signaling^[118]^. Immune cells from the spleen or lungs of patients who died from sepsis have reduced ability to secrete pro-inflammatory and anti-inflammatory factors, overexpression of programmed death receptor (PD-1), and increases in regulatory T-cells (Tregs) and myeloid-derived suppressor cells (MDSCs). It is indicated that both pro-inflammatory and anti-inflammatory responses occur in sepsis patients^[16,119]^. Due to uncontrollable primary infection or acquired infection caused by opportunistic pathogens, dysregulation of immune cells causes immunosuppression or even death^[120]^.

### Excessive inflammation

#### Neutrophils

**Figure 2.**
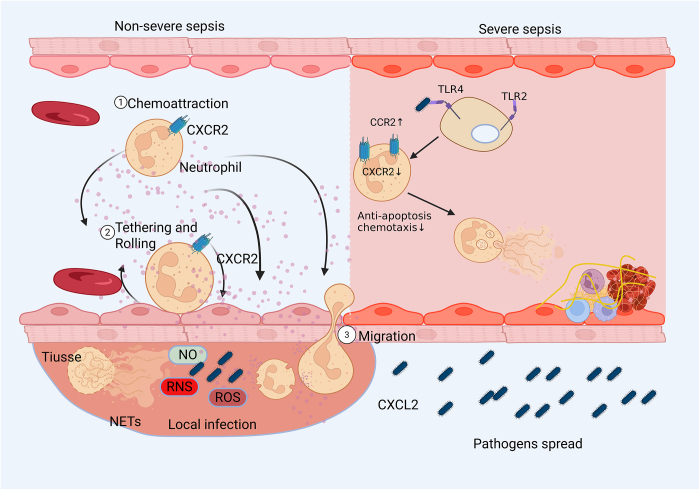
Neutrophil and sepsis. (A) Neutrophils expressing CXCR2 are drawn from blood to the infection site in response to CXCL2 during non-severe sepsis. When neutrophils arrive at the site of infection, they emit antibacterial compounds such as ROS, NO, and NETs, which destroy microorganisms. To stop infections from spreading, lymphocytes and other immune cells might move to the infection site. (B) On the other hand, neutrophils exhibit a longer lifespan and decreased functionality during severe sepsis because of the up- and down-regulation of CCR2 and CXCR2. On the one hand, infections spread as a result of impeded migration. Conversely, a large number of neutrophils are restricted to the vascular endothelium and produce NETs, which cause thrombosis, endothelial damage, and vascular inflammation. CCR2 CC receptor 2; CXCR2 CXC receptor 2; NETs neutrophil extracellular traps; ROS reactive oxygen species; TLR Toll-like receptor; RNS reactive nitrogen species; NO nitric oxide.

In the early stage of sepsis, neutrophils broadly participate in the inflammatory response. Neutrophils expressing CXCR2 are recruited from the blood to the initial site of infection in response to CXCL2^[121]^. Following their migration to the infection site, neutrophils assure host defense by phagocytosis, degranulation and release of neutrophil extracellular traps (NETs)^[122]^. The migration of neutrophils to the site of infection can be impaired during the progression of severe sepsis by activating TLRs. Domer *et al*^[123]^ reported that TLR2 expression on neutrophils is directly activated to downregulate CXCR2 and impair chemotaxis. As a result, neutrophils build up in blood vessels and promote the infection’s spread. Under normal circumstances, neutrophils do not have CC receptor 2(CCR2), but TLR activation causes CCR2 to be expressed in circulating neutrophils (Fig. 2)^[124]^.

**Figure 3.**
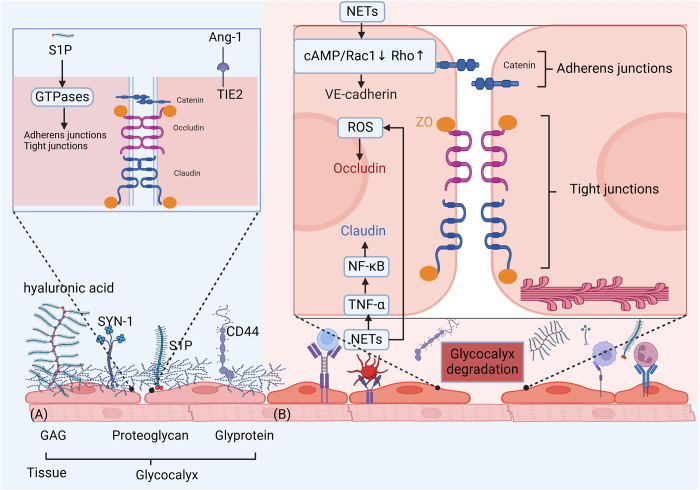
Endothelial dysfunction and sepsis. The glycocalyx of endothelial cells is damaged and the permeability of the endothelium is increased in sepsis (A) NETs can directly degrade the glycocalyx during sepsis by using proteases and hcDNA. Furthermore, glycocalyx can be harmed by a significant variety of inflammatory cytokines, including TNF-α, IL-6, and IL-8. (B) Following the breakdown of the glycocalyx, endothelial cell adhesion molecules become visible, which causes further inflammation, leukocyte and platelet attachment, and rolling. Junction cleavage is the primary cause of the increase in para-endothelial permeability. It is demonstrated that TNF-α activates NF-κB, which disrupts claudin. Occludin redistributes due to ROS, which reduces its ability to bind to ZO-1. VE-cadherin is prone to being broken down by enzymes. NETs use histone and MPO to promote apoptosis. GAG glycosaminoglycan; VE-cadherin vascular endothelial-cadherin; ZO zonula occluding; S1P Sphingosine-1-phosphate; TIE2 Ang-1 receptor; Ang-1 angiopoietin-1; ROS Reactive oxygen species; SYN1 Syndecan-1.

NETs are extracellular fiber networks released by activated neutrophils composed of proteases, histones and DNA^[125]^. NETs are capable of neutralizing and killing bacteria, viruses and fungi, as well as inhibiting their spread^[126,127]^. Destruction of NETs increases bacterial burden and mortality in septic mice, suggesting the importance of NETs in host defense^[128]^. In a state of an imbalanced immune system, excessive NETs aggravate sepsis by inducing an inflammatory response and organ damage^[127,129]^. Abundant neutrophils are released from the bone marrow in response to infections or inflammations, during which the release of proteolytic enzymes, reactive oxygen species (ROS) and reactive nitrogen species (RNS) at the same time further amplifies the inflammatory cascade^[125,130]^. Neutrophils have the shortest lifespan of all types of white blood cells, and this is a favorable condition to prevent excessive inflammation. Most immune cells experience apoptosis during the process of sepsis, forming an immunosuppressive microenvironment. Sepsis, however, offers a microenvironment to delay neutrophil apoptosis, resulting in a prolonged inflammatory response^[131]^. Single-cell RNA sequencing in mice confirmed that LPS-induced overexpressed of programmed cell death ligand 1 (PD-L1) inhibits the immune system, directly triggers lymphocyte apoptosis and finally causes sepsis-associated immunosuppression^[132]^. Excessive NETosis during sepsis is responsible for intravascular thrombosis, DIC and organ failure by tissue damage and overactivation of the coagulation system^[133]^. Histones and DNA fibers form a network in NETs that draws platelets, albumin, plasma proteins, red blood cells, and other components, creating a positive feedback loop that improves thrombus formation both *in vivo* and *in vitro* (Fig. 3)^[134,135]^. Neutrophils also release NETs carrying active tissue factor (TF)^[136]^. High-level TF-rich NETs in sepsis patients are linked with immune thrombosis and poor outcomes^[137]^. NET-induced thrombosis is an established factor for ischemic organ damage and DIC^[138]^. Accumulating evidence has supported the role of the correlation between damage-associated molecular patterns (DAMPs) and NET in sepsis (Fig. 3)^[139]^. Formed by histones and DNA of DAMPs, NETs activate PRRs^[139]^. DAMPs cause NETosis as well. HMBG-1 is found to promote the formation of NETs through TLR4^[139-141]^. Neutrophils promote macrophage pyroptosis in sepsis through NETs and aggravate sepsis-induced acute lung injury^[128,142]^. Moreover, NETs induce the generation of M1 macrophages to release inflammatory cytokines (such as IL-1, IL-6, IL-8, and TNF)^[143]^.

#### Endothelial cells

In sepsis, the coagulation system, vascular endothelium, and activated complement system interact and contribute to the inflammatory response. Vascular endothelial cells are mostly affected by sepsis in the early stage of inflammation and coagulation^[144]^. Endothelial injury increases vascular permeability, allowing fluid to leak from the blood vessels into the interstitial space. Consequently, hypovolemia reduces perfusion pressure and oxygen. Forming as a loss of intravascular volume, hypovolemic shock occurs within hours^[145,146]^.

**Figure 4.**
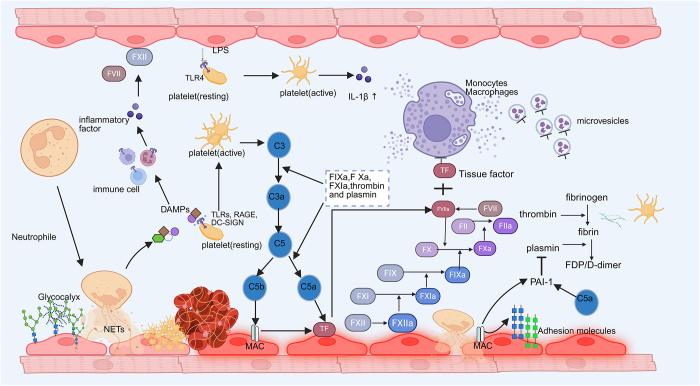
Activation of the coagulation and immune systems. Pathogens stimulate macrophages/monocytes to release cytokines and microvesicles, thereby initiating inflammation and coagulation. Activated neutrophils, by inflammatory cytokines, then expel NETs and result in endothelial cell damage. They also release DAMPs and inflammatory mediators. DAMPs activate the TLRs of immune cells, leading to the release of pro-inflammatory cytokines, which in turn activate coagulation factors VII and XII, initiating the coagulation cascade. Platelets recognize DAMPs through TLRs, RAGE, and DC-SIGN, releasing cytokines (C3) that participate in the inflammatory response. The activated complement system eventually forms the MAC and damages endothelial cells. The extrinsic and intrinsic pathways initiated by tissue factors synergistically activate the coagulation cascade to produce the key mediator thrombin. Further activating inflammation and coagulation, thrombin ultimately stimulates the formation of an inflammatory thrombus. LPS lipopolysaccharide, TLR Toll-like receptor; DAMPs damage-associated molecular patterns, RAGEs receptors for advanced glycation endproducts, DC-SIGN dendritic cell-specific intercellular adhesion molecule-3-grabbing non-integrin, NETs neutrophil extracellular traps, TF tissue factor; MAC membrane attack complex, FVIIa factor VIIa, PAI-1 plasminogen activator inhibitor-1.

Vascular endothelial activation and dysfunction may play a role in sepsis phenotypes and are associated with patient age. Compared with patients younger than 50 years old, those aged 70 and above exhibit relatively mild endothelial activation and dysfunction due to sepsis^[118]^. In a homeostatic state, the endothelial barrier is functionally maintained by the cytoskeleton, glycocalyx, intercellular adhesion molecules, and other support proteins. Tight junctions (TJs) and adherens junctions are two predominant adhesions between endothelial cells. Occludins and claudins, which are linked to the actin cytoskeleton by zonula occludins (ZO), make up TJs (Fig. 4)^[147,148]^. Local infections stimulate the adherence of leukocytes and platelets to endothelial surfaces and further migration to infected sites. Increased para-endothelial permeability in sepsis is mainly attributed to junction cleavage and aggravated by excessive inflammatory responses. Finally, the loss of barrier function increases the mortality of sepsis^[149]^. Rho activity and kinases like Src and Pyk2 are stimulated when the cAMP/Rac1 signaling pathway is inhibited during the inflammatory response triggered by NETs(Fig. 4)^[150]^, both of which can lead to VE-cadherin phosphorylation, vinculin dissociation, and endocytosis^[151]^. Endocytosis of VE-cadherin expands the gap between endothelial cells and thus increases the permeability of the vascular barrier^[152]^. Endothelial barrier failure is caused by the inflammatory cytokine TNF-α, which activates the NF-κB pathway and degrades Claudin-5 at the intercellular junctions of endothelial cells^[153]^. ROS causes occludin to reorganize at intercellular junctions, which facilitates its dissociation from ZO-1 and raises the endothelium’s soluble permeability (Fig. 4)^[154]^. Intravascular proteins and plasma escape into the extravascular space when the barrier is compromised, which decreases tissue edema and microvascular perfusion.

One of the initial signs of endothelial dysfunction in sepsis is the destruction of the endothelial glycocalyx (eGC) structure^[146]^. The endothelial glycocalyx is a layer of carbohydrate-rich material that covers the luminal surface of endothelial cells and provides multiple protective functions to the vascular system^[155]^. Through mediating vascular permeability and endothelial cell response to shear stress, eGC inhibits leukocyte and platelet adhesion, coagulation cascade activation, as well as microthrombi formation^[156]^. Impaired eGC exacerbates endothelial dysfunction and septic complications. An *in vivo* sepsis model demonstrated that sepsis-induced reduction in the thickness of eGC leads to albumin extravasation and increased production of circulating glycocalyx products (e.g., hyaluronic acid and CD44)^[157]^. The potential mechanism can be explained through a series of cascade reactions, in which a disintegrin and metalloproteinase 15 (ADAM15) cleaves CD44 on the eGC, leading to increased concentrations of soluble CD44 and hyaluronic acid in the plasma. Subsequently, this results in a reduction of endothelial barrier integrity and the destruction of vascular endothelial cadherin and β-catenin, which are important components of adherens junctions (Fig. 4)^[157]^. Endothelial TJs and gap junctions are disrupted by sepsis^[158]^. Various eGC products emerge as biomarkers to predict sepsis. Elevated plasma Syndecan-1 levels during sepsis, for example, are associated with the outcome of renal replacement, respiratory failure, and multiple organ dysfunction syndrome (MODS), serving as biomarkers to predict coagulation failure and its mortality^[159-161]^. Sphingosine-1-phosphate (S1P) is a component of eGC that links with vascular leakage and predicts the mortality of sepsis (Fig. 4) ^[162,163]^.

Plasma-derived S1P can activate the S1P receptor 1 on endothelial cells to maintain vascular integrity^[164]^. Angiopoietin-1 (Ang-1) contributes to maintaining vascular integrity via activating the receptor TIE2 (also known as Ang-1 receptor) (Fig. 4). In addition, angiopoietin-2 (Ang-2) is a functional antagonist of Ang-1 that disrupts endothelial barrier function. Its plasma level is an independent predictor of organ dysfunction and death during sepsis^[162,165]^. The predictive performance of Ang-2 in sepsis-related acute kidney injury is superior to soluble endothelial cadherin (sVE-cadherin), endocan and syndecan-1^[163]^. An elevated plasma Ang-2/Ang-1 ratio is believed as an effective biomarker for assessing vascular barrier dysfunction in sepsis patients (Fig. 4)^[166]^.

#### Activation of the complement system

A close interaction exists between the coagulation system and complement system (Fig. 3). Once the infection aggravates to sepsis or septic shock, the complement pathway is deeply activated and is no longer associated with dysregulated inflammatory responses or clinical outcomes. This means that the complement system has been activated to a degree that it no longer has predictive value for clinical outcomes^[167]^. It is known that the complement is activated through three independent pathways: the classical pathway involving antigen-antibody complexes, the alternative pathway involving factors B/D, and the lectin pathway. The formation of C3 convertases is the point at which the three pathways of complement activation converge. PAMPs (e.g., LPS, mannose, and antigen-antibody complexes) and DAMPs are known to activate the classical complement pathway. A cascade of complement cleavage and assembly leads to the formation of catalytic C3 convertase (C4b2b). C3 is then cleaved to obtain excess C3b that amplifies the complement response. The cleavage products of C3 covalently bind to the surface of the pathogen and promote phagocytosis by neutrophils, which is an antimicrobial process specifically referred to as opsonization. C5 forms the C5b-9 complement complex, also known as the membrane attack complex(MAC), which induces cell lysis and clears specific types of intracellular bacteria by creating pores on cells^[168]^. Anaphylatoxins C3a and C5a exert potent pro-inflammatory effects by directly binding to receptors, thus recruiting and activating leukocytes, endothelial cells and platelets^[169]^. Although the activation of the complement system guards protective immunity, an uncontrollable activation causes tissue damage and organ failure^[169]^. Continuous production of C5a paralyzes the innate immune system and weakens the killing effect on bacteria^[170]^. Treatment with C5a receptor antagonists greatly enhances the survival rate of sepsis, whereas the use of C3a receptor antagonists obtains an opposite outcome^[171]^. C5a and C3a exert different roles in the human body. Inhibiting the C5a signaling improves outcomes in mice with polymicrobial abdominal sepsis, mice with sepsis caused by *Streptococcus pneumoniae* infections, and baboons with sepsis caused by *E. coli* infections^[171-173]^. C3a convertase inhibitors not only inhibit the activation of the complement system in sepsis caused by *E. coli* infections, but also attenuate other inflammatory responses, coagulation activation, and multi-organ failure^[174]^.

#### Activation of the coagulation system

Sepsis often accompanies coagulation abnormalities. A multi-center clinical study in Japan found that approximately 61% of sepsis patients suffer from DIC^[175]^. There is an extensive cross-talk between coagulation and inflammation, which may be involved in the pathogenesis of organ dysfunction in sepsis patients^[176,177]^. Activation of the coagulation system in sepsis imbalances and leans towards thrombosis in the microvascular system^[178]^. DIC is the most threatening coagulation dysfunction in sepsis patients, leading to thrombosis and bleeding due to the consumption of coagulation factors, anticoagulant proteins, and platelets. Sepsis-related DIC is mainly caused by thrombosis in microcirculation, eventually leading to organ dysfunction^[178]^. Systemic activation during blood coagulation is one of the dominant pathogenic factors for DIC. Meanwhile, positive expressions of tissue factors on monocytes/macrophages also trigger sepsis-related DIC^[179]^. Pathologically, massive bioactive tissue factors in microvesicles derived from various cell sources contribute to amplifying the coagulation function via binding to activated platelets, neutrophils, and endothelial cells. Factor VIIa (FVIIa) exerts a vital role in sepsis, and its inhibition suppresses LPS- or bacteria-induced activation of the coagulation system in humans and non-human primates^[180]^. Under normal circumstances, the activation of coagulation is controlled by the antithrombin system, activated protein C system and tissue factor pathway inhibitor (TFPI); all three, however, are impaired in sepsis^[180]^.

Platelets are the major blood component responsible for thrombosis and hemostasis. The role of platelets in the pathogenesis of vascular inflammatory processes has been unveiled, such as autoimmune diseases, sepsis, viral infections, and tumor growth and metastasis. Therefore, platelets are considered a type of immune cell involved throughout all stages of the immune response^[181]^. Platelets directly participate in the immune and inflammatory responses via the membrane surface receptors and indirectly recognize PAMPs and DAMPs^[182,183]^ via TLRs^[184-186]^, receptors for advanced glycation endproducts (RAGEs), and dendritic cell-specific intercellular adhesion molecule-3-grabbing non-integrin (DC-SIGN)^[187]^. DAMPs activate the TLRs of immune cells, leading to the release of pro-inflammatory cytokines (such as TNF-α, IL-1, IL-6), which in turn activate coagulation factors VII and XII, initiating the coagulation cascade^[188]^. In addition, platelets are inflammation regulators by directly releasing cytokines (C3, C4a, C1 esterase inhibitor and complement factor H), chemokines and growth factors^[189]^. Cell adhesion molecules are positively expressed on the surface of platelets, thus expanding the antibacterial response of Kupffer cells, neutrophils and the complement system^[186]^. Platelets further promote coagulation and inflammatory responses directly through cell-to-cell contact (leukocytes) and indirectly through the release of proteases and platelet extracellular vesicles (PEVs)^[190]^.

LPS stimulates platelets through the TLR4 pathway, leading to the activation of c-Jun N-terminal kinase and Akt, which ultimately results in an increased secretion of IL-1β in PEVs. The released IL-1β further stimulates the binding of leukocytes to endothelial cells by upregulating VCAM-1^[191]^. Platelets are then responsible for endothelial cell adhesion and extravasation of leukocytes at inflammatory sites, as well as triggering the release of NETs by neutrophils^[192]^. Thromboxane A2 (TXA2) released by neutrophil-platelet aggregates activated endothelial cells through thromboxane receptors, receptor and integrin activation, platelet aggregation, and increased vascular permeability^[193]^. Blocking P-selectin is an effective approach to inhibit the formation of neutrophil-platelet aggregates and reduce plasma TXA2 level, which also greatly prolongs the survival of sepsis-induced mice with acute lung injury via improving alveolar gas exchange^[193]^. Platelets and platelet-derived microparticles expressing phospholipids (e.g., phosphatidylserine) are essential to promote coagulation by increasing the activities of tissue factors FVa and FXa^[190]^.

A bimodal interaction exists between the coagulation system and innate immunity (Fig. 3). Inflammation initiates the coagulation and triggers its activation, and coagulation in turn mediates inflammatory responses^[194]^. Neutrophil elastase, cathepsin G, and nucleosomes altogether inhibit local proteolysis by suppressing the tissue factor pathway. Consequently, the activated coagulation relying on tissue factors and FXII results in coagulation and the formation of intravascular thrombi^[195]^. The interplay between platelets and neutrophils is essential for the excessive inflammation and thrombosis in sepsis. Leukocytes are drawn to the infection site by activated platelets, and they form complexes with neutrophils that lower the neutrophils ‘threshold for releasing NETs and increase the neutrophils’ capacity to kill pathogens (Fig. 3)^[196]^. NETs, released into the vasculature during sepsis, contribute to host defense but can also lead to tissue damage and organ dysfunction. Various components of NETs are also considered to be activators of coagulation^[133]^. NETs-induced intravascular coagulation is dependent on the interaction among histone H4, platelets, and inorganic polyphosphates^[133]^. The extent of NETs formation is a predictive factor for the development and mortality of DIC in sepsis patients, further validating the fundamental role of NETs in sepsis-related coagulopathy^[197]^.

#### Coagulation factors and tissue factors

Proinflammatory signaling is triggered by FVIIa, FXa, thrombin, and fibrin (Fig. 3), mainly by activating protease-activated receptors (PARs) such as PAR1, PAR3, and PAR4^[198]^. Tissue factors mutually activate coagulation proteases. As an illustration, thrombin, FXa, FXa, and C3 transform C3 and C5 into C3a and C5a, respectively; C5a and C5b-9, also referred to as the membrane attack complex, then promote the positive expression of tissue factors on endothelial cells (Fig. 3)^[199]^. C5a interferes with the function of eGC, thus achieving the goal of promoting coagulation. Inflammasomes and gasdermin D (GSDMD) are two mediators for the interaction between inflammation and coagulation. GSDMD causes macrophages and monocytes to undergo pyroptosis via the caspase-1 and caspase-11 enzymes. Released tissue factors in microvesicles derived from pyroptotic cells further activate the coagulation system^[200]^. The function of inflammasomes in the immune and coagulation systems is affected by TMEM173 and HMGB1. TMEM173 is an endoplasmic protein that amplifies inflammation, drives tissue factor release and participates in coagulation reactions by activating GSDMD^[201-203]^. Exogenous HMGB1 forms a molecular complex with LPS and is transported into lysosomes. Macrophages and endothelial cells then result in HMGB1-mediated lysosomal membrane destruction through RAGEs. The released LPS into the cytoplasm activates caspase1-dependent cellular pyrolysis, NLRP3 inflammasomes, and the lethal coagulation pathway^[204]^. Phosphatidylserine-induced GSDMD activation and pore formation are two essential events for tissue factors exposed and activated by HMGB1, rather than GSDMD-induced pyroptosis^[204]^. Caspase-induced cleavage of GSDMD initiates the release of tissue factors, serving as a central stage of DIC that bridges inflammation and coagulation by activated inflammasomes.

### Immunosuppression

**Figure 5.**
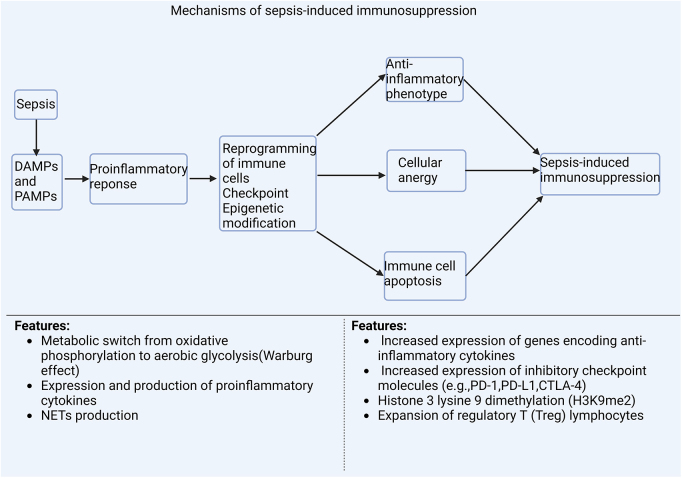
Mechanisms of sepsis-induced immunosuppression. Pro and anti-inflammatory mechanisms make up the host response during sepsis. An infection sets off an initial cytokine-mediated host inflammatory response, which is triggered by an interaction between pattern recognition receptors, PAMPs, and DAMPs and is linked to immune cell reprogramming, checkpoints, and epigenetic modification. Sepsis patients experience excessive inflammatory reactions, which lead to tissue damage and organ failure. At the same time, anti-inflammatory responses are induced and are most likely initiated by inflammation. These responses include cellular anergy and cell death, among other things. The anti-inflammatory milieu persists and sustains immunosuppression. DAMP, damage-associated molecular pattern; PAMP, pathogen-associated molecular pattern; PD-1, programmed cell death protein 1; PD-L1, programmed death ligand 1; ROS, reactive oxygen species.

The extremely high mortality of sepsis is related to hyporesponsiveness (immune tolerance). Immune tolerance is a defense response against inflammatory responses, thus maintaining host homeostasis. Nevertheless, the immune system is unable to recover from the self-induced suppression in sepsis and becomes trapped in a paralyzed state that cannot prevent the progression of infections. Accumulating evidence has highlighted the role of immune cells, immune checkpoints, reprogramming of monocytes/macrophages and epigenetic regulation of gene expressions in sepsis-induced immune tolerance^[17]^ (Fig. 5).

#### Cell apoptosis and release of anti-inflammatory factors

Immunosuppression in sepsis patients is a complicated condition involving multiple types of cells with various characteristics, and linked with the release of anti-inflammatory cytokines, enhanced immune cell apoptosis, T cell exhaustion, immunosuppressive cell hyperproliferation, epigenetic reprogramming and reduced expressions of surface molecules on activated cells^[16,205]^. Immunosuppression in sepsis patients is manifested by an increased susceptibility to secondary infections that are usually caused by opportunistic pathogens and viruses^[206-208]^. Apoptosis of immune cells in sepsis, including CD4^+^ T cells, CD8^+^ T cells, B cells, NK cells and follicular dendritic cells, is activated by the mitochondria (the intrinsic pathway) or the activation of death receptors (the extrinsic pathway)^[209,210]^.

In sepsis patients, there is a selective depletion of memory B cells due to mitochondrial dysfunction^[210]^, a reduction in the expression of major histocompatibility complex class II, and an increase in the expression of the anti-inflammatory cytokine IL-10^[211]^. Inhibiting or preventing apoptosis improves the prognosis in clinical sepsis models, demonstrating the importance of apoptosis in the outcome of sepsis^[212,213]^. Autophagy is a process responsible for maintaining cellular homeostasis via recycling organelles and long-lived proteins. An impaired autophagy is another important event leading to immunosuppression. Lymphocyte-specific loss of Atg5 or Atg7 in mice with abdominal sepsis weakens autophagy, causes immune dysfunction and increases mortality, whereas a T-cell-specific deficiency of Atg5 in sepsis stimulates the secretion of IL-10^[214]^.

The ability of T cells in the blood, spleen, and lungs to produce cytokines is attenuated in sepsis patients^[119,215]^. Moreover, sepsis patients present worse performances in the proliferation of CD8^+^ T cells, cytotoxic function and production of IL-2 and interferon-γ (IFN-γ)^[216]^. The anti-inflammatory response in sepsis is maintained by increased numbers of Tregs and MDSCs, both of which are a mixture of immature myeloid cells that effectively against effector immune cells, especially T cells^[217-219]^. Multiple mechanisms explain the immune dysfunction caused by MDSCs, including deprivation of L-arginine that is essential for T cell function, stimulation of Treg proliferation, and inhibition of macrophage and dendritic cell functions^[219,220]^. The proliferation of MDSCs is correlated with an increased risk of secondary infection in critically ill patients with sepsis^[221]^. Immunocompromised features are observed in neutrophils of sepsis patients, such as migration to multiple chemoattractants, reduced contents of intracellular myeloperoxidase and lactoferrin, and impaired oxidative burst^[132,222]^. Kinase activity in neutrophils is significantly weaker in sepsis patients than in uninfected critically ill patients^[223]^.

#### Immune checkpoints

Immune checkpoints are membrane-bound proteins involved in the immune response as a second signal^[224]^. They are important in sepsis-induced immunosuppression. Programmed cell death-1 (PD-1) is a widely analyzed immune checkpoint in sepsis. Triggering PD-1 on T cells leads to the release of immunosuppressive molecules and ultimately causes apoptosis^[225]^. Overexpression of PD-1 in peripheral blood T cells is associated with inhibited T cell proliferation and increased nosocomial infection and mortality in sepsis patients^[226]^. Blocking PD-1 can effectively improve the prognosis of sepsis in animal models^[227,228]^. A higher expression of PD-1 on blood monocytes, granulocytes, NK cells, T lymphocytes, and B lymphocytes in sepsis patients is associated with a higher mortality^[229-232]^. It is found that (programmed death-ligand 1) PD-L1 and PD-L2 are upregulated on dendritic cells and CD27 + B lymphocytes^[119]^. The expression level of PD-1 on T cells and that of PD-L1 on antigen-presenting cells are associated with lymphopenia and T cell apoptosis in sepsis patients^[119,233]^, but higher expressions of PD-1 and PD-L1 are not correlated with mortality and high risk of nosocomial infection^[231]^. *In vitro*, treatment with anti-PD-1 antibodies suppresses the apoptosis of CD8^+^ T cells and upregulates IFN-γ^[233]^. Considering the correlation of upregulated PD-L1 on neutrophils and monocytes with impaired phagocytic capacity, anti-PD-1 antibodies are found to enhance phagocytosis in leukocytes of sepsis patients^[234]^. It is found that the survival rate of sepsis mice is significantly enhanced by blocking or knocking out PD-1, highlighting the therapeutic efficacy of the PD-1 signaling pathway in sepsis^[228,231]^. PD-1 and PD-L1 and/or PD-L1 pathway inhibitors are expected to reverse immunosuppression, and they have been tested in phase I and II clinical trials for sepsis. Treatment of anti-PD-L1 antibodies presents an acceptable tolerance^[235-238]^, and it increases the absolute lymphocyte count and monocytic human leukocyte antigen-DR isotype (HLA-DR)^[237]^. Inhibition of Treg cell proliferation and PD-1/PD-L1 expression by splenectomy protects mice from sepsis-induced immune cell exhaustion^[239]^. Compared with those of wild-type (WT) mice, PD-1-deficient mice and mice treated with anti-PD-1 antibodies have increased bacterial clearance and reduced bacterial burden^[240]^.

Although a growing number of pieces of evidence have illustrated the role of other immune checkpoints in immunosuppression, their direction function involved in sepsis has been rarely reported^[224,241]^. Cytotoxic T lymphocyte antigen 4 (CTLA-4) is a negative checkpoint on T cells that binds to the B-lymphocyte activation antigens B7-1 (CD80) and B7-2 (CD86). CTLA-4 is overexpressed in CD4^+^ T cells, CD8^+^ T cells and Tregs of mice with intraabdominal sepsis, and treatment with anti-CTLA-4 antibodies greatly enhances mouse survival by reducing sepsis-induced spleen cell apoptosis^[242]^. Moreover, anti-CTLA-4 antibodies also increase the production of IFN-γ in mouse spleen, thereby reducing the mortality of bacterial sepsis caused by lethal cecal ligation and puncture (CLP) and fungal sepsis^[243]^.

#### Reprogramming of immune cells

Impaired reprogramming of monocytes and macrophages is a key feature in immunosuppression. Corresponding events like the reduced production of pro-inflammatory cytokines (also known as LPS tolerance) and downregulation of MHC Class II molecules are responsive to the stimulation by bacterial agonists^[119,244]^. An *in vitro* stimulation weakens the ability of leukocytes in sepsis patients to release pro-inflammatory cytokines (e.g., TNF, IL-1a, IL-6, and IL-12), whereas the ability to release anti-inflammatory mediators (e.g., IL-1 receptor antagonists) is unhurt or even stronger^[245,246]^. Flow cytometry data revealed that a reduced pro-inflammatory response in monocytes, CD4^+^ T cells, CD8^+^ T cells, B cells, and neutrophils isolated from sepsis patients is associated with the downregulation of phosphorylated NF-κB^[247]^. Reprogramming of macrophages is mediated through balancing the equilibrium between p65-p50 and p50-p50 NF-κB pathways in LPS tolerance^[248]^. Anti-inflammatory phenotypes of LPS-tolerant leukocytes are found in the lungs of animals with peritonitis and postmortem sepsis patients^[119,249]^. Cross-tolerance is a phenomenon that occurs when the ability of bacterial agonists to produce pro-inflammatory cytokines is weakened by the stimulation with another agonist. For example, an *in vivo* LPS exposure of human blood leukocytes induces cross-tolerance to reduce the production of TLR2, TLR4, TLR5, and TLR7 receptors^[250]^. DAMPs induce cross-tolerance with LPS, indicating that multiple mechanisms underlying reprogramming of monocytes in sepsis^[251,252]^. Notably, some types of cells in mice have initiated reprogramming without the involvement of cross-tolerance, such as alveolar macrophages, Kupffer cells, microglia, and lymphocytes in the intestinal epithelium and skin^[253]^. Downregulation of monocytic HLA-DR is a biomarker of sepsis-induced immunosuppression and is associated with a high incidence of nosocomial infections and increased mortality^[254,255]^.

#### Epigenetic regulation of gene expression

Epigenetic regulation of gene expression contributes to the reprogramming of immune cells in sepsis, particularly through histone modifications and DNA methylation^[256]^. Chromatin regions are responsible for gene transcription into actively transcribed genes or transcriptionally silent genes^[257]^. Histones form the structure of DNA in chromatin, and prevent DNA from becoming tangled and blocking transcription. Acetylation of lysine residues on the histone is involved in gene transcription, whereas methylation leads to the formation of silent heterochromatin^[257]^. Methylation of histone H3 lysine K4 (H3K4) and histone H3 lysine K27 (H3K27) is highly associated with transcriptional activation and repression, respectively. Downregulation of open chromatin, such as H3K4me3 (histone H3 trimethylated at lysine 4), underlies LPS-induced monocyte tolerance^[258]^. H3K9me2 is a repressive signal that increases in the promoter regions of genes encoding IL-1β and TNF in LPS-resistant macrophages^[259,260]^. A similar finding is also yielded in LPS-resistant blood monocytes from sepsis patients^[260]^. Upregulation of histone demethylase KDM6B (also known as JMJD3) by activated NF-κB^[261]^, and inhibition of transcription genes due to accumulated protein deacetylase sirtuin-1 on the promoters of TNF and IL-1β explain the epigenetic mechanism of LPS in affecting gene transcription^[262]^. Single-cell RNA sequencing of peripheral blood monocytes and dendritic cells revealed a unique population of CD14^+^ monocytes with an immunosuppressive phenotype, named MS1. Downregulation of MHC class II molecules, activation of NF-κB and reduced production of TNF are found in LPS-induced MS1 cells^[263]^. Overall, single-cell sequencing provides insights into distinct cellular phenotypes of either pro-inflammatory or immunosuppressive in sepsis^[263]^.

Infections cause long-lasting epigenetic changes. IL-12 derived by dendritic cells is long-term downregulated for 6 weeks and longer after the original septic event in mice, which is attributed to the transcription of IL-12 p35 and IL-12 p40 influenced by histone modifications^[264]^. A long-term epigenetic change following pneumonia results in the resistance of macrophages and impaired functions to phagocytose antigens and bacteria^[265]^. In mice, epigenetic modifications are brought about by sepsis. When inflammatory gene promoters have defective H3K4me3 (activation mark) at NF-κB-binding sites, mice with recovered abdominal sepsis have downregulated Mixed-lineage leukemia 1 in their bone marrow-derived macrophages^[266]^. Long-term peripheral macrophage function degradation is caused by epigenetic alterations that begin in bone marrow progenitor stem cells after post-sepsis^[266]^. Collectively, immunosuppression is a persistent condition in newly proliferating leukocytes in sepsis and is associated with long-term recurrence and mortality.

### Gut microbiota

Gastrointestinal dysfunction, intestinal dysbiosis, bacterial translocation and destruction of intestinal homeostasis are frequently observed in sepsis patients. The intestinal mucosal barrier damage further promotes the release of inflammatory factors, and the vicious circle eventually exacerbates sepsis^[267]^. As a result, the destruction of intestinal homeostasis and intestinal mucosal barrier are both consequences and initiators of sepsis^[268]^. Commensal bacteria are present in the gut and are essential for maintaining homeostasis and protecting the host from pathogenic bacterial invasion. Physiologically, gut microbiota directly or indirectly resists colonization and invasion by harmful microorganisms. Additionally, host cells manufacture antimicrobial proteins for them. For instance, commensal (LPS and flagellin) microbe-associated molecular patterns (MAMPs) stimulate epithelial cells and dendritic cells through TLR-mediated means, resulting in the production of the islet-derived protein IIIγ by Paneth cells. The interaction between other intestinal bacteria and intestinal epithelial cells increases IgA produced by B lymphocytes, induces Th17 cell differentiation and produces pro-inflammatory factors^[269,270]^. Metabolites of gut microbiota influence the function of immune cells. Pathogens entering the circulation system are usually captured and killed by Kupffer cells. D-lactic acid produced by gut microbiota is transferred to the liver through the portal vein, thus influencing the response of Kupffer cells to capture and kill pathogens^[271]^. Short-chain fatty acids (SCFAs) are the main metabolites produced by microorganisms, showing pleiotropic effects on the host through driving monocyte differentiation into macrophages, inhibiting histone deacetylase-3, and regulating the barrier function of intestinal epithelium^[268]^. Butyrate produced by *Clostridium* and *Faecalibacterium* is a regulator of colonic T cell differentiation by upregulating Foxp3, and it also reduces the expression levels of inflammatory factors (TNF-α, IL-6) by inhibiting histone deacetylation^[272]^. In addition, the dominant gut microbiota can directly inhibit those entering the intestine by competing for nutrients and producing virulence factors (antimicrobial peptides)^[269,270]^.

Disruption of the gut microbiota enhances disease susceptibility. A large-scale observational study provided indirect evidence for sepsis caused by the impaired gut microbiota^[273-275]^. Alterations in the gut microbiota result in the increased permeability of the intestinal barrier and pathogen spreading throughout the body^[276]^. Moreover, the host’s ability of remote organs to defend against infections, such as bone marrow and lungs, is weakened following the disruption of the gut microbiota^[268]^. Prospective cohort studies have demonstrated an association between lower diversity of gut microbiome and higher abundances of pathogenic bacteria in sepsis (e.g., aerobic Gram-negative bacteria)^[277-279]^. Higher abundances of *Cronobacter* and *Cronobacter phage* in patients with sepsis-associated myocardial injury than in sepsis patients without tissue injury are indicative of the prognostic value of gut microbiota^[280]^. High-sensitivity cardiac troponin T and N-terminal pro-B-type natriuretic peptide have become indicators for the diagnosis and prognosis of sepsis-associated myocardial injury^[281,282]^. Critically ill patients with systemic inflammatory response syndrome (SIRS) have lower abundances of obligate anaerobes but higher abundances of potential pathogens like *Staphylococcus* and *Pseudomonas*^[283]^. Deshmukh *et al* verified the role of gut microbiota in regulating the homeostasis and number of neutrophils in neonatal mice with sepsis of *E. coli* infection^[284]^. An increased alpha diversity in the gut microbiome via co-housing in turn increases CD4^+^ T cell response and survival of septic mice^[285]^. Metabolites derived from gut microbiota are important mediators of liver, lung, kidney and cardiac function^[286,287]^. SCFAs are a typical type of metabolites produced by gut microbiota, which extensively participate in immune response, inflammatory response, and carbohydrate and lipid metabolism^[288,289]^. Through providing essential energy to intestinal epithelial cells in the colon, butyrate contributes to the regulation of intestinal gene expressions and suppression of LPS-induced intestinal inflammation^[290]^. Zhan *et al* summarized the capacity of SCFAs to defend against pathogen invasion as energy sources and regulators of barrier function and immune status of the host intestine^[291]^. SCFAs also reduce the expression levels of pro-inflammatory factors (e.g., NO, TNF-α, IL-6, IL-1β, IL-10) by mediating the production of immune cytokines^[292,293]^, and influence immune cell function through post-transcriptional modifications in the acute phase of infections. In mice, acetate promotes the acetylation of GAPDH to catalyze glycolysis by supplementing acetyl-CoA, thereby promoting the rapid recall response of CD8^+^ T cells and immune responses^[294]^.

In summary, the significance of gut microbiota in the context of sepsis should not be underestimated or overlooked. Before the onset of sepsis, gut microbiota are altered to increase the susceptibility to sepsis through increasing the abundances of pathogenic bacteria, priming the immune system to produce a robust pro-inflammatory response, and reducing the production of beneficial microbial products. Once sepsis attacks the human body, the disruption of gut microbiota further worsens and causes a higher risk of Multiple Organ Dysfunction Syndrome (MODS). There is limited evidence that microbiome-based therapeutics, including the use of probiotics and selective decontamination of the digestive tract, can reduce the risk of sepsis and improve the outcomes, although safety concerns.

## Treatment of sepsis

At present, there lack of effective targeted therapies for critically ill patients with sepsis. Current management of sepsis mainly focuses on controlling the infection source (surgical removal and/or use of antibiotics), stabilizing hemodynamics (hydration, use of vasoactive drugs in patients with septic shock) and regulating the host response^[18,295]^. Organ replacement therapies, such as renal replacement therapy and mechanical ventilation, are required to relieve organ dysfunction during sepsis. Unfortunately, effective drugs to block or disrupt pathophysiological pathways involved in the development of sepsis are not available. Despite pouring massive medical resources into rescuing sepsis, the mortality of sepsis remains as high as 25%-30%, or even 50% in septic shock patients^[296,297]^.

Sepsis is a clinical syndrome^[298]^. Patients with a history of previous disabilities or chronic diseases are more prone to develop sepsis. Infection is the root cause of sepsis responsible for initiating and sustaining immune dysregulation. Early identification, early antibiotic treatment, and early source control of infection are crucial for the prognosis of sepsis. Early source of infection control may require surgical or invasive procedures to manage the source of infection^[18,299]^. Source control refers to the implementation of single or combined measures to eliminate the focus of infection or control the factors of infection, including pathogen detection, early anti-infective treatment, and removal of the causes of infection. For patients with early suspected infection, timely pathogen detection should be performed. After confirmation of the diagnosis, it is crucial to identify the site of infection and take appropriate control measures. Measures for source control of infection include the use of surgical methods to eliminate the source of infection and to control ongoing infection. These measures are based on debridement, drainage, decompression, and restoration of anatomical function^[300,301]^. Taking intra-abdominal infection as an example, all guidelines point out that thorough control of the source of infection at the first moment is fundamental to the treatment of intra-abdominal infection. Once intra-abdominal infection is complicated by organ dysfunction and sepsis, it is necessary to establish a multidisciplinary team for comprehensive treatment of intra-abdominal infection under the leadership of surgery^[301,302]^.

An early antibiotic treatment is preferred for sepsis. The *SSC Guidelines 2021* recommended an intravenous infusion of one or more broad-spectrum antibiotics within 1 hour of the diagnosis of sepsis. The mortality of patients with septic shock receiving an antibiotic treatment within 3 hours significantly increases by 35% for every hour of delay in antibiotic infusions^[303]^, whereas the increased mortality is not observed in sepsis patients without confirmed shock^[304]^. However, the continuous narrowing of the antibiotic prescription window compels medical personnel to sacrifice diagnostic accuracy in pursuit of speed^[305]^, inevitably leading to the overuse of antibiotics^[306]^. Before the recognition of a certain pathogen, an early empiric antibiotic therapy provides clinical benefits to sepsis with an unknown source of infections^[307]^. A retrospective cohort study in the United States in 2014 showed that the dominant bacteria for pediatric sepsis include *Staphylococcus aureus*, methicillin-resistant *Staphylococcus aureus, fungi, Pseudomonas*, and *Clostridium difficile*^[308]^. The bacterial spectrum of sepsis has changed within the past decade. An empiric antibiotic therapy for sepsis is recommended to target these suspected pathogens^[308]^.

Intravenous fluid resuscitation is a key component of the initial resuscitation of sepsis^[309]^. As early as 2001, an early goal-directed treatment was proposed to lower the mortality of severe sepsis^[310]^. The initial fluid resuscitative goal is to optimize hemodynamics to achieve a blood oxygen saturation (SO_2_) of >70% in the superior vena cava (SVC), central venous pressure (CVP) of 8-12 mmHg, mean arterial pressure (MAP) of ≥65 mmHg, and urine output of ≥0.5 mL/kg/h, which is expected to reduce the incidence of MODS and in-hospital mortality of sepsis. Nevertheless, data from multi-center randomized studies reported no survival benefits of goal-directed fluid resuscitation^[311]^, and increasing the medical cost^[312,313]^. Consequently, the *Third International Consensus Definitions for Sepsis and Septic Shock* highlighted an expert consensus process based on the clinical practice of sepsis and septic shock^[3]^. The time-to-treatment initiation and types and volume of infused fluid have been modified in the fluid resuscitation for sepsis. Fluid resuscitation is applied to individuals with hypotension, elevated lactic acid levels, or oliguria. At present, a new sepsis “hour-1 bundle” has been introduced to the management of sepsis, that is, fluid resuscitation is initiated within 1 hour of diagnosis of sepsis^[314]^. An optimal type of fluid infused to sepsis patients needs to have similar chemical compositions, be metabolized and completely excreted from the body without producing metabolic or adverse events but enhancing the intravascular volume. If the fluid has a high economic benefit in improving the prognosis of sepsis, then so much the better. A randomized controlled trial that compares the influence of balanced crystalloid solution versus 0.9% sodium chloride on 30-day hospitalization mortality of sepsis concludes that the better outcome of the former^[315]^. Adding an appropriate volume of colloid solution to the crystalloid fluid not only reduces the total volume of infused fluid but also lowers the risk of edema in sepsis patients^[316]^. The SSC guidelines recommended the infusion of crystalloids at a rate of 30 mL/kg (actual body weight) within the first 3 hours, for 2000 mL of crystalloid fluid total^[18]^. The hour-1 bundle protocol for sepsis is designed to ensure early and maximal correction of hypotension. Hemodynamic monitoring on 71 sepsis patients found no significant differences in the 24-hour urine output and 28-day mortality between the treatment of crystalloid infusion at 30 mL/kg versus <30 mL/kg^[317]^. The prognosis of the 3-hour bundle therapy is related to the subtype of sepsis^[318]^. Therefore, we suggested an individualized fluid resuscitation based on close monitoring of clinical data for sepsis. A series of methods are available to achieve an accurate monitoring of systemic hemodynamics, such as bedside ultrasonography, chest impedance, pulmonary artery catheterization, and use of a pulse index continuous cardiac output. Dynamic variables are used clinically to assess fluid responsiveness, mainly including parameters obtained from the passive leg raise and fluid replacement test, pulse pressure variation, stroke volume variation and respiratory variation in the diameter of the inferior vena cava^[319,320]^.

Sepsis resuscitation bundle, although being placed at the core of treatment, is controversial. Evidence has claimed that the hour-1 bundle does not improve the prognosis of sepsis, but causes a high risk of over-medication. Three randomized controlled trials of ARISE, ProMISe, and ProCESS all firmly demonstrated a similar outcome between the sepsis resuscitation bundle versus traditional treatment^[313,321,322]^. Antibiotics are key to treating sepsis, and the choice and timing of antibiotic use greatly influence the outcome of sepsis. Misuse of antibiotics increases the risk of drug resistance and alters the composition of gut microbiota^[18]^. The treatment of sepsis is complicated and challenging. An individualized precision treatment has become mainstream for sepsis.

## Endotypes/phenotypes of sepsis and novel therapeutic approaches

For sepsis, existing drugs do not work. Sepsis is a heterogeneous disease, with varying pathogenic organisms (such as Gram-positive or Gram-negative bacteria, viruses, or fungi), origin organs (such as lungs, urinary tract, or abdomen), and host immunological characteristics (ranging from hyperinflammation to immunosuppression) among patients^[323]^. It is unrealistic to expect a single drug to modulate the distorted immune response across the entire disease spectrum.

A drug that is effective for one person may even exacerbate the condition of others, for example, when patients with immunosuppression receive immunosuppressive drugs, or when patients with hyperinflammation receive immunostimulatory drugs. Therefore, precision medicine is a disease treatment approach in which patients are stratified based on clinical and biological criteria, as well as biomarkers (including immune, laboratory, high-resolution imaging, and omics-based measurements) to identify patterns, thereby formulating more precise prevention, diagnosis, and treatment strategies that take into account individual medical and health characteristics^[324]^. To successfully implement precision medicine for sepsis, a promising approach is to classify sepsis into different subtypes and provide corresponding treatments; it is necessary to predict and enrich the prognosis for sepsis patients^[325,326]^. Current research on sepsis subtypes is still in its preliminary stages, and there are no unified standards regarding inclusion criteria and research methods. Based on different classification foundations, sepsis subtypes (Table 2) are mainly divided into the following categories: (1) sepsis endotypes based on genomic indicators; (2) sepsis subtypes based on biomarkers and clinical indicators; (3) sepsis phenotypes based on clinical big data. Since endotypes and subtypes originate from different patient characteristics, they cannot be easily linked.

**Table 2:** Phenotypes of sepsis

	Year	Phenotypes & manifestations	Ref.
Clinical data	2015	Phenotypic clusters within sepsis-associated multiple organ dysfunction syndrome, including the shock with elevated creatinine, minimal MODS, shock with hypoxemia and altered mental status, and hepatic disease.	^[327]^
	2016	Clinical phenotypes of sepsis are classified by liver function and coagulation function, including the non-hepatobiliary dysfunction/DIC group and the hepatobiliary dysfunction/DIC group.	^[328]^
	2018	Four phenotypes of sepsis (α, β, γ, and δ). The α phenotype is the most common phenotype of sepsis with the use of the lowest dosages of vasopressor drugs; The β phenotype is usually diagnosed in older patients and those with more comorbidities or renal insufficiency; The γ phenotype is manifested with inflammatory responses and respiratory insufficiency; The δ phenotype is the most lethal, resulting in high risks of hepatic insufficiency and shock, and high lactate levels.	^[329]^
	2018	Four subphenotypes of sepsis (profile 1-4). Profile 1 is characterized by relatively mild symptoms and the lowest mortality; Profile 2 is characterized by respiratory dysfunction; Profile 3 is characterized by MODS and shock, and the highest 90-day mortality and hospital mortality; Profile 4 is characterized by neurological dysfunction.	^[330]^
	2019	Four subphenotypes of sepsis divided by temperature trajectories, including the hyperthermic, slow resolvers (14.9% of the cohort); hyperthermic, fast resolvers (23.2%); normothermic (32.8%); and hypothermic (29.1%).	^[331]^
Biomarkers	2019	Sepsis is divided into the MALS group and the non-MALS group, and ferritin can be used as a biomarker for an early diagnosis of MALS.	^[332]^
	2022	Sepsis is classified into MALS, immunoparalysis and intermediate based on biomarkers of ferritin >4420 ng/mL and <5000 HLA-DR receptors/monocytes.	^[333]^
	2023	Glycerophospholipids are the distinct metabolic patterns in sepsis-induced ARDS, and the sphingolipid metabolism distinguishes sepsis-induced direct from indirect ARDS subphenotypes.	^[334]^
Genomics	2009	Sepsis is classified into subclasses A, B, and C based on genome-wide expression profiling. Subclass A is associated with downregulated genes related to adaptive immunity and glucocorticoid receptor signaling pathways, resulting in more severe conditions and higher mortality.	^[335]^
	2013	Based on the prognostic enrichment analysis of proteomics-metabolomics data, the high mortality of sepsis is associated with fatty acid transport, β-oxidation, gluconeogenesis, and citric acid cycle. Profiles of proteins and metabolites change in sepsis, and cis-4-decenoylcarnitine, 2-methylbutyrylcarnitine, butyrylcarnitine, hexanoylcarnitine, lactate, age, and hematocrit are 7 biomarkers predicting the mortality of sepsis.	^[336]^
	2016	Two distinct SRSs, SRS1 and SRS2, are determined by cluster analysis on transcriptional profiles of leukocytes in peripheral whole blood.	^[337]^
	2017	Four molecular endotypes for sepsis designated Mars1-4, are divided based on genome-wide blood gene expression profiles. Mars1 endotype is characterized by the worst condition, slow innate immunity and adaptive immunity, highest risk of shock and highest mortality. BPGM and TAP2 genes are specific for the diagnosis of Mars1 endotype. Mars3 endotype is characterized by the lowest risk of mortality, showing an RNA expression pattern consistency with the increased adaptive immunity and T cell function.	^[338]^
	2018	Sepsis is classified into three subtypes inflammopathic (characterized by innate immune activation and higher mortality), adaptive (characterized by adaptive immune activation and lower mortality), and coagulopathic (characterized by platelet degranulation, coagulopathy, higher mortality and older age) based on RNA expressions.	^[339]^
	2020	A total of 1613 sepsis patients are classified into Class 1 (characterized by immunosuppression and higher mortality) and Class 2. A 5-gene class model involving C14orf159, AKNA, PILRA, STOM and USP4 is created and validated in an external cohort, showing a better performance than the SRS endotypes and a similar performance as the APACHE II score.	^[346]^
	2022	RAI in rodents is an endotype of sepsis characterized by a persistent high inflammatory response. Glucocorticoids are beneficial to sepsis with RAI, but harmful to those without RAI.	^[379]^
	2023	Targeted metabolomics identified 14 metabolites to distinguish sepsis-induced ARDS subphenotypes, mainly including the lysophosphatidylethanolamine (lysoPE) plasmalogen, PE plasmalogen and phosphatidylcholine.	^[334]^

APACHE II, acute physiology and chronic health evaluation II; ARDS, acute respiratory distress syndrome; DIC, disseminated intravascular coagulation; DR isotype; HLA-DR, human leukocyte antigen – SRSs, sepsis response signatures; MALS, macrophage activation-like syndrome; MODS, multi-organ dysfunction syndrome; RAI, relative adrenal insufficiency.

The treatment of sepsis based on precision medicine proposes specific therapies for sepsis. Zhang *et al* analyzed clinical data of 14 993 sepsis patients available from the Medical Information Mart for Intensive Care III database. Through cluster analysis of medical data within 24 hours of admission, sepsis patients are classified into 4 subclasses and found that sepsis patients of different subtypes had different responses to fluid therapy. Profile 3 is characterized by a lowered mortality after fluid infusion, whereas profile 4 presents the opposite result^[330]^. Based on sepsis biomarkers, precision medicine-based immunotherapy is proposed. Circulating biomarkers, such as C-reactive protein, ferritin, IL-6, and soluble urokinase plasminogen activator receptor (suPAR), are capable of identifying excessive inflammation in sepsis and dividing sepsis into the macrophage activation-like syndrome (MALS) group and non-MALS group^[332]^. Immunoparalysis is one endotype of sepsis featured as lymphopenia, impaired production of cytokines and low expression of HLA-DR on monocytes^[119,340]^. So far, targeted therapies for MALS (e.g., rIL-1Ra/anakinra)^[333,341]^ and immunoparalysis (e.g., rIFNy, IL-12, GM-CDF, rIL-7, and checkpoint inhibitors) have been applied to sepsis patients with certain endotypes/phenotypes^[236,342]^. Small-scale phase 2 randomized trials have been performed to illustrate the efficacy and safety of sepsis treatment based on the endotype/phenotype classification^[333]^.

Genome testing of the whole blood in sepsis patients provides useful information to reflect the immune state and guide clinical treatment. Divided by the sepsis response signatures (SRSs), SRS1 is manifested as an immunosuppressive pattern consisting of endotoxin tolerance, T cell exhaustion, and downregulation of class II HLA molecules. SRS1 causes a higher mortality than SRS2^[337,343]^. In a cohort involving 83 SRS1 patients and 86 SRS2 patients treated with vasoactive drugs versus hydrocortisone, increased mortality in SRS2 patients with a normal immune function is linked with the use of corticosteroids^[344]^. At the same time, considering that SRS1 is similar to pediatric septic shock type A, both suggest immune suppression. Corticosteroid treatment is associated with an increased mortality rate in septic shock type A, but it is not related to SRS1^[335,344]^. Exposure to corticosteroids is associated with increased mortality in adults with endotype A septic shock^[345]^. Zhang *et al* analyzed clinical data of 1613 Chinese sepsis patients from the online databases of the Gene Expression Omnibus (GEO) and ArrayExpress and classified them into Class 1 (characterized by immunosuppression and higher mortality) and Class 2 (increased mortality after the use of hydrocortisone)^[346]^.

At present, precision treatment for sepsis not only involves subtype grouping treatment but also has scholars positioning precision treatment in immunotherapy. With the in-depth study of the pathogenesis of sepsis, it has been found that the disorder of inflammatory immune responses is harmful to the host and is related to organ dysfunction, making immunotherapy a new direction in the effort to treat sepsis. The current immunotherapy for sepsis mainly focuses on cellular regulatory factors, immune checkpoint inhibitors, anti-apoptosis, and the promotion of immune cell hyperactivity. With continuous exploration, a large number of in *vivo* and in *vitro* studies have used immunomodulatory drugs to treat the immunoparalysis condition in sepsis patients.

These treatment methods include popular PD-1/PD-L1 blockers, granulocyte colony-stimulating factors (G-CSF), granulocyte-macrophage colony-stimulating factors (GM-CSF), and INF-γ^[235,333]^. In recent years, with the significant achievements of mesenchymal stem cells (MSCs) in cancer and their proven ability to regulate inflammation, enhance tissue repair, and clear pathogens in sepsis animal experiments, reducing mortality, they have become a new research focus for the treatment of sepsis, currently mainly concentrating on phase 1 safety studies. A summary of these drugs can be found in Table 3.

**Table 3:** Immunomodulatory drugs for immunoparalysis

Immunomodulatory Drugs	Registration No.	Clinical trial phase	Period (year)	Case number	Primary endpoints	Outcomes	State	Ref.
GM-CSF	NCT00252915	2	2005-2007	38 adults were assigned to an intervention group (*n* = 19) and the placebo group (*n* = 19)	Time of mechanical ventilation, Length of stay in hospital/ICU, reconstitution of monocytic immunity	28-day follow-up showing GM-CSF in shortening the time of mechanical ventilation and length of stay in hospital/ICU.	Completed	^[347]^
GM-CSF	NCT02361528	2	2015-2018	98 adults were assigned to the intervention group (*n* = 54) and the placebo group (*n* = 44)	Number of patients presenting at least one ICU-acquired infection at D28 or ICU discharge number of patients presenting at least one ICU-acquired infection at D28 or ICU discharge	ICU-acquired infection during 28-day follow-up. GM-CSF does not affect the prevention of ICU-acquired infection in sepsis immunosuppression.	Completed	^[348]^
rh-GCSF	NCT01479114	4	2009-2010	90 newborns were assigned to the intervention group (*n* = 30), placebo group (*n* = 30) and control group (*n* = 30)	All cause mortality	12-day observation showed less use of antibiotics and a shorter length of stay in the intervention group than placebo. Rh-GCSF improves neutrophilic count and functions as an adjuvant therapy for neonatal sepsis.	Completed	^[349]^
GM-CSF/INF-γ	NCT01374711	Not applicable	2011-2011	18 healthy adults intravenously injected with LPS (1st and 6th day of two cycles) and assigned into the INF-γ group (*n* = 6), GM-CSF group (*n* = 6) and placebo group (*n* = 6)	The effects of GM-CSF/IFN-γ on the development of in vivo immunoparalysis induced by experimental human endotoxemia	IFN-γ upregulates monocytic HLA-DR, but GM-CSF downregulates it (no significant difference versus placebo group). IFN-γ partially reverses immunoparalysis.	Completed	^[350]^
Tα1	NCT00711620	Not applicable	2008-2010	361 adults assigned in Tα1 group (*n* = 181) and placebo group (*n* = 180)	Immune response to Thymosin alpha 1	Higher monocytic HLA-DR on the 7th day in the Tα1 group.	Completed	^[351]^
Tα1	NCT02867267	3	2016-2020	1089 adults assigned in Tα1 group (*n* = 542) and placebo group (*n* = 547)	Incidence of new onset infection within 28 days	The trial did not find conclusive evidence that Tα1 can reduce the 28 – day all – cause mortality rate in adults with sepsis.	Completed	^[352]^
rhIL-17(CYT107)	NCT02640807	2	2016-2018	27 adults assigned into high-dose CYT107 group (*n* = 9), low-dose CYT107 group (*n* = 8) and placebo group (*n* = 10)	CYT107 effect on absolute Lymphocyte Count	Increased lymphocytes at 28 days of CYT107 treatment.	Completed	^[353]^
Anakinra or INF-γ	NCT03332225	2	2017-2019	36 adults were assigned to the intervention group (*n* = 15) and the placebo group (*n* = 21)	Mortality, time to decrease of SOFA score by more than 50%	Mortality does not decrease at 28 days in the intervention group, whereas the Sequential Organ Failure Assessment (SOFA) score decreases at 7 days.	Completed	^[333]^
Anakinra or rhINF-γ	NCT04990232	2	2021-to date	Not applicable	Mean total Sequential Organ Failure Assessment score	The mean SOFA score at 9 days is assessed.	On-going	^[354]^
PD-1/PD-L1 inhibitor (BMS-936 559)	NCT02576457	1	2015-2017	24 adults were assigned to the intervention group (*n* = 20) and the placebo group (*n* = 4)	Immune system function based on baseline and post-dosing assessments of mHLA-DR expression on monocytes at planned sampling timepoints	High-dose BMS-936559 upregulates monocytic HLA-DR	Terminated	^[236]^
PD-1/PD-L1 inhibitor(nivolumab)	NCT02960854	1	2016-2018	31 adults were assigned to the high-dose group (*n* = 16) and the low-dose group (*n* = 15)	Monocytic HLA-DR expression	Upregulated monocytic HLA-DR in both groups	Completed	^[235]^
PD-1/PD-L1 inhibitor(nivolumab)	JapicCTl-173 600	1/2	2017-2018	13 adults were assigned to the high-dose group (*n* = 8) and the low-dose group (*n* = 5)	Lymphocyte count, monocytic HLA-DR expression	Absolute lymphocyte counts and monocytic HLA-DR increase over time in both groups.	Completed	^[237]^
Anti-TNF-α antibody (AZD9773)	NCT01144624	2	2010-2011	20 adults were assigned to the high-dose group (*n* = 7), low-dose group (*n* = 7) and placebo group (*n* = 6)	Pharmacodynamic Effects of AZD9773 on TNF-alpha	AZD9773 decreases TNF-α level at 29 days of follow-up	Completed	^[355]^
β-glucan	NCT01727895	Not applicable	2013-2013	15 healthy adults were assigned to the intervention group (*n* = 10) and the placebo group (*n* = 5)	Production of cytokines, the leukocyte capacity to phagocytose and kill the fungal pathogen *Candida Albicans* (antifungal activity).	7-day follow-up showing no significant influences of oral β-glucan on cytokine production by leukocytes and bactericidal activity	Completed	^[356]^
MSCs	NCT02328612	1	2014-2015	32 healthy adults intravenously injected with LPS and assigned into the intervention group (*n* = 24) and placebo group (*n* = 8)	Inflammatory response as measured by laboratory measurements and functional assays of innate immunology	Intravenous infusion of human adipose MSCs (4 × 10^6^ cells/kg) exerts proinflammatory, anti-inflammatory, and procoagulant effects during human endotoxemia.	Completed	^[347]^
MSCs	NCT02421484	1	2015-2018	9 septic shock patients infused with MSCs at varying doses	Number of adverse events as a measure of safety and tolerability	Infusion of freshly cultured allogeneic bone marrow-derived MSCs at a dose of 3 million cells/kg (250 million cells) into patients with septic shock is safe.	Completed	^[358]^
MSCs	NCT05283317	2	2018-2019	30 adults were assigned to the intervention group (*n* = 10) and the control group (*n* = 20)	Mortality, length of stay in the hospital	Deaths occur in the control group at 1 week, and intervention group at 15 and 28 days during the 28-day follow-up. MSCs positively influence the survival of early-stage sepsis.	Completed	^[380]^
MSCs	NCT04961658	1	2021-2023	Not applicable	The safety of GEM00220 will be assessed by monitoring adverse events	28-day follow-up for the safety.	Completed	^[381]^

anti-TNF-αantibody, anti-tumor necrosis factor-alpha antibody; GM-CSF, granulocyte-macrophage colony-stimulating factor; INF-γ, interferon-gamma; MSCs, mesenchymal stem cells; LPS, lipopolysaccharide; PD-1, programmed cell death protein 1; PD-L1, programmed death-ligand 1; rhIL-17, recombinant human Interleukin-17; rhINF-γ, recombinant human Interferon-gamma; rh-GCSF, recombinant human granulocyte colony-stimulating factor; Tα1, thymosin alpha 1.

The treatment of sepsis is complicated and challenging. This complexity is particularly evident when considering advanced interventions like immunotherapies. For instance, while targeting the PD-1/PD-L1 pathway is a promising strategy to reverse sepsis-induced immunosuppression, the clinical application of PD-1/PD-L1 inhibitors has yielded heterogeneous outcomes. The observed heterogeneity in patient responses to PD-1/PD-L1 inhibitors in sepsis trials highlights the complexity of applying these immunotherapies^[225,235]^. Several factors likely contribute to this variability. A crucial determinant is the patient’s specific immune status at the time of intervention; checkpoint blockade is hypothesized to be most beneficial in individuals exhibiting clear signs of T-cell exhaustion and a dominant PD-1/PD-L1-mediated immunosuppressive phenotype^[212,359]^. Conversely, patients in a hyperinflammatory state, or those whose immunosuppression is driven by alternative mechanisms, may not respond favorably or could even experience adverse effects^[360]^. The timing of administration relative to the evolving immune trajectory of sepsis is therefore critical^[361]^. Furthermore, differences in the infectious pathogens – including their type (e.g., Gram-negative vs. Gram-positive bacteria, fungi, viruses), load, and virulence factor profiles – can profoundly influence the nature and extent of immune dysregulation, including the engagement of the PD-1/PD-L1 pathway^[362]^. The site of primary infection can also shape the local and systemic immune environment, potentially impacting therapeutic efficacy^[363]^. Additionally, patient-specific factors such as comorbidities, genetic predispositions, and the overall severity of sepsis and degree of organ dysfunction likely play a role. Understanding these sources of heterogeneity is paramount for developing predictive biomarkers and tailoring PD-1/PD-L1 inhibitor therapy to the septic patient subgroups most likely to derive clinical benefit, a key tenet of advancing precision immunotherapy in sepsis.

In addition to the aforementioned immune targets, some scholars are dedicated to studying the impact of hormones on the prognosis of clinical patients. Critically ill patients who have been treated in the intensive care unit for a long time may develop relative central adrenal insufficiency^[364]^. Glucocorticoid resistance and hyperlactatemia lead to the fatal septic shock^[365]^. When using small doses of hormones, cellular immunity is mainly suppressed; at high doses, humoral immune function is suppressed by inhibiting plasma cells and antibody production. Glucocorticoids can also alleviate systemic inflammatory responses and tissue damage by inhibiting the production of inflammatory factors. The use of glucocorticoids (GCs) as monotherapy for sepsis remains a controversial issue^[366]^. In 2018, two large-scale randomized controlled trials were published to assess the impact of GCs on patients with sepsis or septic shock, but no clear conclusions could be drawn. These two studies recruited a total of 5041 patients, and the ADRENAL trial (Corticosteroid Therapy for Septic Shock and Adrenal Insufficiency) showed that hydrocortisone treatment did not improve the 90-day survival rate^[367]^. In contrast, the APROCCHSS trial suggested that the treatment of human septic shock with activated protein C and corticosteroids significantly reduced the mortality rate from 49.1% to 43%^[368]^. Metabolic resuscitation with glucocorticoids, along with vitamins C and B1, did not reduce long-term mortality rates^[369]^. A meta-analysis found that corticosteroid administration was associated with reduced 28-day mortality in sepsis patients compared with placebo use or standard supportive care. Given the conflicting conclusions regarding corticosteroid treatment for sepsis, more research is needed in future studies to correlate sepsis subtypes with corticosteroid efficacy and identify subtypes that will benefit from corticosteroid therapy^[370]^

Sepsis still lacks a specific cure, and Uckun *et al* have utilized existing technology to research new drugs. The two formulations of the sepsis candidate drug Rejuveinix (RJX) have been confirmed to have a protective effect in mice^[371]^. Kukoamine B mesylate has been tested in a phase IIa trial for treating sepsis^[372]^. Wang *et al* designed new drugs for treating sepsis and septic cardiomyopathy based on the nanotechnology^[373]^. Current evidence suggests that acupuncture combined with conventional therapy may be beneficial for sepsis compared to conventional therapy alone. Due to the low certainty of its impact, further research is needed^[374]^.

## Precision therapy and future perspectives

The incidence and high mortality rate of sepsis impose a heavy burden on society. Existing drugs can provide support for controlling the source of infection and improving organ function, but there is still no specific cure. Considering the complexity of the host response during sepsis and the diversity of pathophysiological pathways involved^[323]^, the “one-size-fits-all” strategy for treating sepsis is no longer satisfactory.

To better understand sepsis and seek more effective treatment options, we have summarized the pathogenesis and etiology of sepsis, from bacterial invasion (Gram-negative bacteria), drug resistance to the body’s inflammatory response (activation of immune cells, release of inflammatory factors, and activation of the coagulation and complement systems). An uncontrolled cytokine storm can lead to multiple organ failure and even death. Dysbiosis is a factor that clinicians may easily overlook. A low diversity of the gut microbiota and an increased relative abundance of pathogenic Gram-negative bacteria and enterococci can increase the risk of sepsis.

As our understanding of the pathogenesis and pathophysiology of sepsis becomes clearer, we are also making new expectations for its treatment, pushing for precision treatment to replace the “one-size-fits-all” approach. To achieve precision treatment, many scholars have used various means, including multi-omics and analysis of clinical big data, to classify sepsis into different subtypes in an attempt to find appropriate treatment options. Among these approaches, the combination of traditional treatment and immunotherapy has shown promising results in patients with sepsis. For example, the use of immunomodulatory drugs (such as PD-1/PD-L1, G-CSF, GM-CSF) favors sepsis patients with immunoparalysis by increasing the number and function of immune cells and upregulating monocytic HLA-DR. However, it has to be acknowledged that the combination of traditional sepsis treatment strategies and immunotherapy strategies has not significantly improved the mortality rate of sepsis, and clinical trials are also in phase 2–3. For example, T α1 has not improved the 28-day mortality rate of sepsis patients^[352]^. When reviewing these clinical trials, we must consider the following issues: First, the issue of reasonable experimental design. When studying such a complex syndrome as sepsis, is it necessary to consider patient subgroups? At present, in clinical drug research, all sepsis patients are directly divided into treatment and non-treatment groups, and the experimental results show that immunotherapy drugs cannot improve patient prognosis. However, in the clinical and basic research of sepsis, it has been found that immunotherapy may only be effective for patients with a higher risk of death from sepsis^[375]^, while the use of anti-inflammatory drugs for patients with a lower risk of death may increase the mortality rate^[376]^. Second, the site of infection and the pathogen are also important factors. The pathogenic mechanisms after the entry of pathogens into the human body and the host’s immune response are not the same. When we try to improve the prognosis of sepsis, we may need to classify it according to the site of infection and the pathogen, rather than widely including patients in a complex syndrome.

With the rapid development of medical techniques, precision cancer treatment affords us lessons in sepsis treatment^[377]^. The treatment of sepsis will be carried out in a pathologically specific and time-dependent manner, rather than based on clinical manifestations. To achieve precision treatment, many scholars have used various means to detect changes within the body that divide sepsis into different endotypes/phenotypes. We envision that in the future, personalized treatment plans can be formulated for septic patients. The clinic also needs to conduct a comprehensive assessment and testing of each patient’s immune function to provide precision treatment plans, such as giving targeted pro-inflammatory or anti-inflammatory treatments according to the patient’s immune status. Therefore, precision treatment strategies oriented towards clinical subtypes-immune status-drug/immunotherapy may be the next major advancement in the treatment of sepsis.

However, there are still some difficulties to overcome in achieving precision treatment for sepsis. Currently, there is no relevant research/mechanism that breaks down the barriers between various endotypes/subphenotypes, and there is no clear correlation between any subtype classification methods. Some scholars have proposed that a disease axis approach could be used to divide sepsis into different subtypes, but no specific conclusions have been drawn yet^[378]^. Therefore, there is an urgent need to establish a classification scheme for subtypes that associates the pathogenesis, pathophysiology, and target organ localization of sepsis with disease subtypes and immune status, providing clear references for clinical practice. It is hoped that in the future, we can utilize well-reproducible, timely laboratory tests or simple bedside tests to quickly and accurately determine the pathophysiological changes and disease status within the body during sepsis, guiding individualized and precision treatment for different patients.

## Data Availability

This study relies on previously published data.
